# Protocol for dynamic high-throughput cell death screening of primary phagocytes following microplastic and nanoplastic exposure

**DOI:** 10.1016/j.xpro.2025.104204

**Published:** 2025-11-14

**Authors:** Tim Leonardus Philip Skrabanja, Joëlle Anna Zoetje Klazen, Sarah Sayed, Loes Leonie Francisca Heeren, Giulio Giustarini, Nienke Vrisekoop

**Affiliations:** 1 Department of Respiratory Medicine, Center of Translational Immunology, University Medical Center Utrecht, Lundlaan 6, Mail Room KC02.085.2, 3584 EA Utrecht, the Netherlands

**Keywords:** cell biology, high throughput screening, immunology, microscopy, molecular biology

## Abstract

Micro- and nanoplastics (MNPs) can cross epithelial barriers of the lung and/or intestine into the bloodstream. In the body, phagocytes will be exposed to plastic particles, but they are incapable of degrading them. Here, we present a protocol for high-throughput cell death screening of primary phagocytes following MNP exposure. We describe steps for isolating primary phagocytes, plating these with MNPs, and time-lapse imaging. Further, we explain detailed procedures for image analysis using the IncuCyte S3 live-cell imaging system and analysis software.

For complete details on the use and execution of this protocol, please refer to Giustarini et al.^1^

## Highlights

• Instructions for measuring particle-induced cell death of neutrophils and monocytes
• Detailed guidance of isolating neutrophils and monocytes to prevent activation
• Step-by-step explanation of the Incucyte data analysis and masking procedures
• Different effects of polystyrene and silica particles on innate immune cell viability

## Abstract (editor highlights)

Publisher’s note: Undertaking any experimental protocol requires adherence to local institutional guidelines for laboratory safety and ethics.

## Graphical abstract

**Figure:**
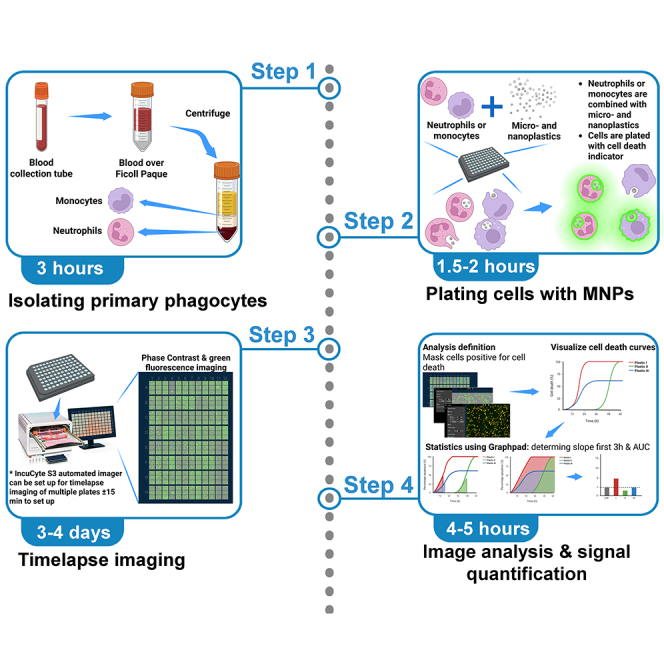

## Before you begin

Since the 1950s, plastic use has increased enormously which resulted in widespread pollution in the environment.^2^ Plastics from the environment can break down into smaller fragments which are so-called micro- and nanoplastics (MNPs). Humans are continuously exposed to MNPs from the environment. MNPs can cross the epithelial barriers of the lung and/or intestine so that they can enter the blood circulation and become systemic.^3^^,^^4^ Unlike biological pathogens, MNPs cannot be degraded and cleared by the immune system. Innate immune cells and especially neutrophils and monocytes are the first responders in case a pathogen enters the body. Although direct consequences of MNP exposure on human health are unknown, it has already been found that MNPs are associated with toxicity in monocyte-derived dendritic cells, macrophages and T-cells and lead to immune dysregulation.^5^^,^^6^^,^^7^^,^^8^^,^^9^

Here, we describe a high-throughput cell death assay to dynamically screen MNP particle toxicity on human neutrophils and monocytes with the Incucyte® S3 Live-Cell Analysis System. MNP toxicity is measured as the fluorescent signal area from dead cells, stained for AnnexinV-FITC and or SytoxAADvanced, followed over 72 h. The Incucyte S3 microscope allows you to visually screen cell death for multiple conditions at regular time intervals without the need to manually focus.

This protocol explains the procedures required for the isolation and handling of neutrophils and monocytes from Ficoll isolation and to prepare experimental conditions and buffers. We also provide steps on how to apply the Incucyte S3 settings for the experimental set-up. Finally, we explain instructions on how to analyze the generated Incucyte S3 data in R and GraphPad.

Here, we describe cell death screening for monodisperse polystyrene (PS) and silicon dioxide (SiO_2_ or silica) particles of 0.2, 1 and 10 μm in diameter. We describe the protocol for PS particles because they are representative microplastic test particles because of their widespread application in *in vitro* and *in vivo toxicity* testing and their wide commercial availability.^1^^,^^10^ We also include silica to show that this dynamic high-throughput screening is also suitable for non-plastic particle toxicity testing. The aim of this protocol is to enable other laboratories to reproducibly screen multiple plastic (or non-plastic) particles for toxicity-induced cell death in primary innate immune cells.

**CRITICAL:** Before you begin, check if your imaging-plate is compatible with the Incucyte S3 microscope. The Incucyte S3 microscope is calibrated for a wide array of commonly used plates but an incorrect plate that is not recognized can result in trouble with automatic focusing because the system cannot find the correct Z-position of the plate bottom.

**CRITICAL:** All experimental steps need to be performed under sterile conditions to avoid the risk of contamination which may affect the outcomes of the experiment. We perform all experimental steps described in this protocol in a biosafety cabinet in a BSL-1 facility.

**CRITICAL:** Primary human neutrophils are very sensitive and short-lived cells which become quickly activated due to incorrect handling procedures. Activated neutrophils have a shorter live span compared to non-activated neutrophils. Therefore, we recommend to perform Ficoll layering and use the described PBS2+ wash buffer and HEPES3+ culture medium instead of other isolation methods or cell culture media to minimize neutrophil cell death.

**CRITICAL:** For normal neutrophil cell functioning, it is crucial that whole blood is obtained in sodium heparin collection tubes instead of EDTA or sodium citrate collection tubes because these anti-coagulators chelate Ca^2+^ ions. Cells require Ca^2+^ for functioning and its reduction by chelation has negative effects, especially on neutrophils.^11^

**CRITICAL:** Ca^2+^ is also essential for binding of AnnexinV-FITC to phosphatidyl residues in death cells. Make sure that your culture medium contains at least 1–3 mM Ca_2_Cl.

**CRITICAL:** Check in advance if the particles you want to test show autofluorescence or that dyes bind to the particles. Potential autofluorescence or unwanted particle staining may interfere with the fluorescent signal from death cells which affect the analysis.

***Note:*** Check in advance the (effective) density of the particles you want to test. If the particles have a lower buoyancy than the culture medium, the cells at the bottom of the well cannot interact with the particles. In that case, you are still able to investigate the effects of potential leachates of chemicals that leak out of the plastics particles.

### Innovation

The innovation of this protocol is that it describes the use of the Incucyte S3 microscope for particle toxicity testing instead of soluble compound toxicity testing. Here, we describe a method to perform dose-response toxicity testing for plastic particles via microscopy assessment of the cells. The advantage of the Incucyte S3 is that you can visually assess the cells and their interaction with the particles at regular intervals without the need to manually switch between multiple conditions or to focus on your cells. In advance, you adjust the desired settings in the Incucyte S3 software and it will automatically screen your imaging plate. Compared to LDH or MTT assay toxicity testing, you can visually observe what happens to your cells which makes the testing less of a ‘black box’. In case something goes wrong in the experiment, you can directly see this in your results from the Incucyte S3 images. Based on the experience of our lab, we also describe the application and preparation of supplemented HEPES3+ culture and supplemented PBS2+ wash buffers as preferred neutrophil media, in contrast to regular used supplemented RPMI 1640 medium or PBS, which are not the optimal cell medium or wash buffer for primary neutrophils.

### Institutional permissions

Neutrophils and monocytes were isolated from blood from healthy participants that were recruited from the staff and students via the “Mini Donor Service” at the University Medical Center Utrecht, the Netherlands (Biobank number 18-774). Healthy participants were between 18–65 years old and included both sexes. Approval from the medical ethics committee was obtained and all participants provided written informed consent in accordance with the declaration of Helsinki. Users of this protocol should obtain the acquired ethical requirements from their institutions to work with human blood and in a BSL-1 facility.

### Preparation of ammonium-chloride-potassium lysis buffer

**Timing: 1 h**

Ammonium-Chloride-Potassium-(ACK-)lysis buffer removes the erythrocytes during the neutrophil isolation using Ficoll layering via lysis. Here, the protocol describes how to prepare it in steps 1–7.

Ammonium-Chloride-Potassium-(ACK-)lysis buffer consists of 155 mM NH_4_Cl, 10 mM KHCO_3_, 0.1 mM Na_2_EDTA, Phenol Red diluted in ddH_2_O. Add these compounds as described in steps 1a-e. ACK-lysis buffer can be made in large batches and stored at 4°C for a year.

1. Preparation of ACK-lysis buffer.
  a. Add 0.4 L Milli-Q water to an autoclaved 0.5 L glass bottle.
  b. Weigh 4.15 g NH_4_Cl (155 mM).
  c. Weigh 0.5 g KHCO_3_ (10 mM).
  d. Weigh 18.6 mg Na_2_EDTA (0.1 mM).
  e. Weigh 0.2 g phenol red.

**CRITICAL:** Autoclave your bottle in advance to make sure that it has cooled down before use.

***Optional:*** The phenol red is not necessary for the ACK-lysis buffer but helps you in assessing the pH.

2. Add the components from step 1b-e to the 0.4 L Milli-Q water.
  a. Mix by inverting the solution a few times.
3. Add 0.1 L Milli-Q water to the bottle until 0.5 L of buffer.
  a. Mix the solution on a magnetic stirrer at 4°C for 15 min.

**CRITICAL:** Always check if the compounds are dissolved, otherwise continue with stirring as described in step 3a.

4. Set the pH of the buffer at 7.4 at 4°C (this is the equivalent of pH 6.9 at 20°C).

***Optional:*** Increase the pH by adding droplet for droplet a solution of 1.25 M NaOH or decrease the pH by adding droplet for droplet a solution of 37% HCl. Both NaOH and HCl are dissolved in Milli-Q water.

5. Measure the osmolarity of the ACK-lysis buffer with an osmometer. The osmolarity should be between 295–309 mOsm.

**CRITICAL:** The osmolarity should never exceed 310 mOsm.

***Note:*** In our lab, we used a 3320 Osmometer to measure osmolarity.

***Note:*** The osmolarity can be reduced by adding more Milli-Q water and increased by adding more of the ACK-lysis buffer mixture.

6. Sterilize the ACK-lysis buffer with a 0.22 Steritop Filter in a BSL-1 biosafety cabinet.
  a. Sterilize the outside of the glass bottle containing unfiltered ACK-lysis buffer with a 70% EtOH solution before placing it in the biosafety cabinet.
  b. Sterilize the outside of an autoclaved empty 0.5 mL glass bottle with 70% EtOH and add it in the flow cabinet underneath the Steritop filter.
  c. Turn on the vacuum suction system in the biosafety cabinet.
  d. Transfer the unfiltered ACK-lysis buffer to the Steritop filter to filter it into the empty glass bottle.

**CRITICAL:** Check in advance if the flow cabinet has a vacuum suction system for the filtering of the ACK-lysis buffer.

**CRITICAL:** Although the glass bottles are autoclaved, there is a risk for contamination during the weighing of the compounds and adjusting the pH or osmolarity. Therefore, never skip filtering the ACK-lysis buffer.

**CRITICAL:** When transferring the buffer to the Steritop filter, perform the steps always under sterile BSL-1 conditions in a biosafety cabinet to prevent contamination.

7. Store ACK-lysis buffer at 4°C for up to 6 months or until the color of the phenol red changes from pink to yellow/orange.

**CRITICAL:** If the pink color of the ACK-lysis buffer changes to yellow, the pH has dropped, and the buffer cannot be used for erythrocyte lysis. Perform steps 1–6 for new buffer preparation.

### Preparation of supplemented PBS buffer (PBS2+)

**Timing: 30 min, performed under sterile conditions in a BSL-1 biosafety cabinet**

PBS2+ buffer is required to dilute whole blood before Ficoll layering as described in step 1 in the step-by-step method details section. PBS2+ buffer functions also as wash buffer for ACK-lysis buffer during neutrophil or monocyte isolation. It consists of Phosphate Buffered Saline (PBS) containing two additional components. Add Albuman (human serum albumin, stock 40 g/L) to a concentration of 4 g/L and add trisodium citrate (TSC) to a concentration of 0.32% w/v to PBS to prepare PBS2+ wash buffer. All steps in the buffer preparation and storage are performed under BSL-1 conditions in a biosafety cabinet as described in steps 8–9.

***Note:*** This buffer can be made in a large batch and stored in 40 mL aliquots at 4°C for 1 week or at −20°C for 1 year.

8. Preparation of 3.2% w/v TSC.
  a. For 1 L PBS2+ wash buffer, add 10.67 mL 30% w/v TSC to 89.33 mL Milli-Q water to make 100 mL 3.2% w/v TSC.
9. Preparation of PBS2+ wash buffer.
  a. Add 100 mL 3.2% w/v TSC from step 8a and 100 mL Albuman (human serum albumin, stock 40 g/L) to 800 mL PBS.
  b. Mix well before aliquoting by inverting the solution.
  c. Aliquot the PBS2+ wash buffer to 40 mL in 50 mL tubes and store them at −20°C for later use.

**CRITICAL:** Keep the PBS2+ wash buffer and its compounds sterile.

### Preparation of supplemented HEPES solution (HEPES3+)

**Timing: 1 h**

HEPES buffer consists of 20 mM HEPES, 132 mM NaCl, 6 mM KCl, 1 mM MgSO4, 1.2 mM KH_2_PO_4_ in MilliQ. HEPES buffer can be made in large batches and stored in 40 mL aliquots at 4°C for one week or at −20°C for one year. Supplemented HEPES3+ is the optimal culture medium for primary neutrophils. The preparation is described in step 10–15.

10. Preparation of HEPES buffer.
  a. Add 1 L of MilliQ-water to a one-liter autoclaved glass bottle.
  b. Weigh 4.77 g HEPES.
  c. Weigh 7.71 g NaCl.
  d. Weigh 0.45 g KCl.
  e. Weigh 0.25 g MgSO_4_·7H_2_O (246.47 g/mol).
  f. Weigh 0.16 g KH_2_PO_4_.

**CRITICAL:** Autoclave your bottle in advance to make sure that it has cooled down before use.

11. Add the components to the 1 L Milli-Q water.
  a. Mix by inverting the solution a few times.
12. Set the pH of the HEPES buffer to 7.4 at 21°C.

***Note:*** Use a pH meter or testing strips of your choice to test the pH of the HEPES buffer.

***Optional:*** Increase the pH by adding droplet for droplet a solution of 1.25 M NaOH or decrease the pH by adding droplet for droplet a solution of 37% HCl. Both NaOH and HCl are dissolved in Milli-Q water.

13. Measure the osmolarity with an Osmometer.
  a. The osmolarity of the HEPES buffer should be 280 mOsm.
  b. The osmolarity can be reduced by adding more MilliQ and increased by adding more of the HEPES buffer mixture.
14. Sterilize the buffer by passing it over a 0.22 μm Steritop filter in a BSL-1 biosafety cabinet.
  a. Sterilize the outside of the glass bottle containing unfiltered HEPES buffer with a 70% EtOH solution before placing it in the biosafety cabinet.
  b. Sterilize the outside of an autoclaved empty 0.5 mL glass bottle with 70% EtOH and add it in the flow cabinet underneath the Steritop filter.
  c. Turn on the vacuum suction system in the biosafety cabinet.
  d. Transfer the unfiltered HEPES buffer to the Steritop and filter it into the empty glass bottle.
  e. Aliquot the HEPES buffer to 40 mL in 50 mL tubes and store them at −20°C for later use.

**CRITICAL:** Check in advance if the flow cabinet has a vacuum suction system for the filtering of the HEPES buffer.

**CRITICAL:** Although the glass bottles are autoclaved, there is a risk for contamination during the weighing of the compounds and adjusting the pH or osmolarity. Therefore, never skip filtering the HEPES buffer.

**CRITICAL:** Perform steps 14a-e always under sterile conditions in a flow cabinet at BSL-1 conditions to prevent contamination.

15. Supplement the prepared 40 mL HEPES buffer as described in the previous steps 10–14 with three extra components to make HEPES3+ culture buffer for neutrophils.
  a. Thaw a 40 mL HEPES buffer aliquot.
  b. Prepare a 2.5 M D-(+)-Glucose solution in MilliQ-water and add 80 μL of this sterile glucose solution to the 40 mL HEPES aliquot.
  c. Prepare a 100 mM CaCl_2_ solution in MilliQ-water and add 400 μL of this sterile CaCl_2_ solution to the 40 mL HEPES aliquot.
  d. Add 1 mL sterile Albuman 200 g/L (human serum albumin) to the 40 mL HEPES aliquot.

**CRITICAL:** The addition is performed under BSL-1 conditions in a biosafety cabinet. Make sure that the glucose and CaCl_2_ stocks in MilliQ are kept sterile or filter them over a 0.22 μm Steritop filter.

**CRITICAL:** Add the sterile glucose, CaCl_2_ and Albuman 200 g/L (human serum albumin) to the HEPES buffer shortly before use in the experiment. You can store the HEPES3+ at 4°C up to a week but we recommend keeping it as freshly prepared as possible since you are not able to freeze it after the addition of the Albuman. Our group does not use HEPES3+ older than a week.

***Note:*** You can prepare and aliquot 100 mM CaCl_2_ and 2.5 M D-(+)-Glucose solution in MilliQ-water in advance under sterile BSL-1 conditions in a biosafety cabinet. Filter both with a 0.22 μm Steritop Filter before aliquoting.

### Preparation of supplemented RPMI 1640 cell culture medium

**Timing: 30 min, all steps are prepared under BSL-1 conditions in a biosafety cabinet**

Supplemented RPMI 1640 with 2 mM L-glutamine is used as basis for the adherence of monocytes after the Ficoll step, washing away non-adherent peripheral blood mononuclear cells (PBMCs) and preparing the experimental conditions.

For these three purposes different amounts of HPS are added. This will be described in steps 16a-d.

***Note:*** There is no need to use or prepare supplemented RPMI 1640 if you plan on only isolating neutrophils for your experiment.

***Note:*** As alternative to RPMI 1640 containing phenol red. You can also use Gibco RPMI 1640 Medium, no phenol red, in case phenol red interferes with your imaging.

16. Prepare RPMI 1640 with 2 mM L-glutamine as basis for monocyte incubation medium and monocyte wash medium.
  a. Add 5 ml of a 200 mM L-glutamine to 500 mL RPMI 1640.
    **CRITICAL:** Use this medium to prepare your experimental conditions. Additional HPS is added during the preparation of the experimental steps.
  b. Supplement RPMI 1640 with added L-glutamine from step 16a with 10% human pooled serum (HPS) as adherence medium for the PBMCs during 3-h incubation, to adhere monocytes to the 96-wells imaging plate described in step 3 under monocyte isolation.
    i. Take 36 mL of supplemented RPMI 1640 with L-glutamine as prepared in step 16a and add 4 mL HPS (40 mL total).
  c. Supplement RPMI 1640 with added L-glutamine from step 16a with 1% human pooled serum (HPS) to prepare washing medium to remove non-adherent PBMCs after 3-h incubation.
    i. Take 39.6 mL of supplemented RPMI 1640 with L-glutamine as prepared in step 16a and add 0.4 mL HPS (40 mL total).
  d. You can store the supplemented RPMI 1640 with L-glutamine and without or 1 or 10% HPS at 4°C for three months.
    **CRITICAL:** Do not use this medium with additionally added 10 or 1% HPS to prepare your experimental conditions. Otherwise, the amount of HPS will exceed 10% in the experiment.

### Preparation of 0.5% Triton X-100 in PBS

**Timing: 30 min, all steps are prepared under BSL-1 conditions in a biosafety cabinet**

Although you strive to add the same volume of neutrophils or monocytes per well of the imaging-plate, the number of cells in the field of view may vary and some conditions, for example control or conditions with low or non-toxic particles may not reach maximum cell death after 72 h. To achieve maximum cell death in all conditions, you need to add 0.5 % Triton X-100 in PBS to all conditions after at least 72 h of imaging. Triton X-100 disrupts the cellular membrane and kills the remaining living cells. Here, the protocol describes how to prepare it.

**CRITICAL:** It is essential to obtain the maximum value of cell death prior normalization of the data. Otherwise, you cannot compare the different conditions with each other.

17. Preparation of 0.5% Triton X-100 solution in PBS.
  a. Add 40 mL of PBS to a 50 mL tube.
  b. Cut the pipet tip of a 1 mL tip with scissors.
  c. Take up 200 μL Triton X-100 stock with the cut pipet tip and add to the 40 mL PBS.
  d. Vortex thoroughly to completely dissolve the Triton X-100.
  e. Store the 0.5% Triton X-100 solution at 4°C for 3 months under sterile conditions.

**CRITICAL:** Prepare the 0.5% Triton X-100 solution in advance of the experiment.

**CRITICAL:** Pipetting Triton X-100 can be difficult due to its viscosity. Therefore, we recommend cutting the tip of the 1 mL pipet tip for better pipetting. There is a chance that the added Triton does not dissolve immediately in the PBS and forms flakes. It helps to prewarm the PBS at 37°C and vortex the solution after addition.

## Key resources table

**Table:** 

REAGENT or RESOURCE	SOURCE	IDENTIFIER
**Biological samples**		

Human blood of healthy volunteers age 18–65	Mini Donor Service, University Medical Center Utrecht, the Netherlands	Biobank number 18-774

**Chemicals, peptides, and recombinant proteins**		

FITC Annexin V	BioLegend, San Diego, USA	Cat#640906; RRID: AB_2561292
SYTOX AADvanced Dead Cell Stain Kit	Thermo Fisher Scientific	Cat#S10349
Human Serum Type AB (male) from male AB (non-heat-inactivated) (human pooled serum/HPS)	Sigma-Aldrich	Cat#H4522-100ML
Albuman 200 g/L (human serum albumin)	Prothya Biosolutions Netherlands B.V. Amsterdam, the Netherlands	08717185830897
Albuman 40 g/L (human serum albumin)	Prothya Biosolutions Netherlands B.V. Amsterdam, the Netherlands	08717185830835
Trisodium citrate 30% w/v (per mL: 300 mg HOC(COONa)(CH2COONa)_2_ · 2H_2_O, 1.16 mmol citrate and 3.43 mmol sodium)	Pharmacy Spaarne Gasthuis. Haarlem, the Netherlands	Cat#S219.1
Aqua (MilliQ-water)	B. Braun Medical B.V. Oss, the Netherlands	Cat#10005844
Dulbecco’s phosphate-buffered saline (PBS)	Sigma-Aldrich	Cat#D8537
HEPES	Sigma-Aldrich	Cat#H3375
Sodium chloride (NaCl)	Sigma-Aldrich	Cat#S9888
Potassium chloride (KCl)	Sigma-Aldrich	Cat#P9327
Magnesium sulfate (MgSO_4_·7H_2_O)	Sigma-Aldrich	Cat#63138
Monopotassium phosphate (KH_2_PO_4_)	Sigma-Aldrich	Cat#P0662
Calcium chloride (CaCl_2_)	Sigma-Aldrich	Cat#499609
D-(+)-glucose	Sigma-Aldrich	Cat#G8270
Ammonium chloride (NH_4_Cl)	Thermo Fisher Scientific	Cat#15121993
Potassium bicarbonate (KHCO_3_)	Thermo Fisher Scientific	Cat#30006931
Ethylenediaminetetraacetic acid disodium salt dihydrate (Na_2_EDTA)	Carl Roth GmbH + Co KG	Cat#8043.1
Phenol red	Sigma-Aldrich	Cat# 1072410005
Gibco RPMI 1640 medium	Thermo Fisher Scientific	Cat#12017599
Gibco RPMI 1640 medium, no phenol red	Thermo Fisher Scientific	Cat#11530406
L-Glutamine Solution 200 mM	Thermo Fisher Scientific	Cat#59202C
N-Formyl-L-methionyl-L-leucyl-L-phenylalanine (f-MLF)	Sigma-Aldrich	Cat#F3506
Polybead Microspheres 0.20 μm particles (2,7% w/v) (2,700 mg/mL)	Polysciences GmbH, Germany	Cat#07304-15
Polystyrene 1 μm particles (10% w/v) (100 mg/mL)	Microparticles GmbH, Berlin, Germany	Cat#PS-F-KM508-2
Polystyrene 10 μm particles (10% w/v) (100 mg/mL)	Microparticles GmbH, Berlin, Germany	Cat#PS/Q-R-KM618
Silica 1 μm particles (5% w/v) (50 mg/mL)	Microparticles GmbH, Berlin, Germany	Cat#SiO2-R-SC278-2
Silica 10 μm particles (5% w/v) (50 mg/mL)	Microparticles GmbH, Berlin, Germany	Cat#SiO2-R-SC222-2
Ficoll Paque Plus	Cytiva	Cat#17-1440-03
Triton X-100	Sigma-Aldrich	Cat#T8787

**Software and algorithms**		

IncuCyte Software v2022RevB	Sartorius	www.sartorius.com
GraphPad Prism version 10.04.0 (621)	GraphPad Software	www.graphpad.com
RStudio version [1] ‘2024.12.0.467’	R Project	https://www.r-project.org/

**Other**		

9 mL VACUETTE sodium heparin blood collection tube	Greiner Bio-One, Kremsmünster, Austria	Cat#455051
0.22 μm Merck Steritop Quick Release	Merck MilliPore	Cat#15770319
Black/clear polystyrene flat bottom imaging 96-well plate (Corning/Falcon)	Corning	Cat# 353219
Incucyte® S3 Live-Cell Analysis Instrument	Sartorius	https://www.sartorius.com/en/products/live-cell-imaging-analysis/live-cell-analysis-instruments/s3-live-cell-analysis-instrument
Hettich Rotanta 460 Centrifuge 5650	Hettich Rotanta	https://www.hettichlab.com/products/centrifuges/benchtop-centrifuges/rotanta-460-460-r/
CELL-DYN Emerald Hematology Analyzer	Abbott	https://www.corelaboratory.abbott/us/en/offerings/brands/cell-dyn/cell-dyn-emerald.html

## Materials and equipment

**Table:** ACK-lysis Buffer

Reagent	Concentration	Amount
Preparation of ACK buffer		
NH_4_Cl	155 mM	4.15 g
KHCO_3_	10 mM	0.5 g
Na_2_EDTA	0.1 mM	18.2 mg
Phenol Red	N/A	0.2 g
MilliQ-water	N/A	500 mL
**Total**	**N/A**	**500 mL**

***Note:*** ACK-lysis buffer should be stored at 4°C for 6 months.

**Table:** 3.2% w/v Trisodium Citrate for Phosphate Buffered Saline (PBS)2+ wash buffer

Reagent	Final concentration	Amount
Preparation of 3.2% w/v Trisodium Citrate for PBS2+ wash buffer		
Trisodium citrate 30% w/v (per mL: 300 mg HOC(COONa)(CH2COONa)_2_ · 2H_2_O, 1.16 mmol citrate and 3.43 mmol sodium)	3.2% w/v	10.67 mL
MilliQ	N/A	89.33 mL
**Total**	**3.2% w/v**	**100 mL**

***Note:*** Prepare 3.2% w/v TSC just before preparing the PBS2+ wash buffer itself.

**Table:** Phosphate Buffered Saline (PBS)2+ wash buffer

Reagent	Final concentration	Amount
Preparation of PBS2+ wash buffer		
Albuman 40 g/L (human serum albumin)	4 g/L	100 mL
Trisodium citrate 3.2% w/v in MilliQ	0.32% w/v	100 mL
Phosphate Buffered Saline (PBS)	N/A	800 mL
**Total**	**N/A**	**1000 mL**

***Note:*** Aliquots of PBS2+ can be stored as 40 mL aliquots at 4°C for 1 week or at −20°C for 1 year.

**Table:** HEPES buffer

Reagent	Concentration	Amount
Preparation of HEPES-buffer		
HEPES	20.0 mM	4.77 g
NaCl	132.0 mM	7.71 g
KCl	6.0 mM	0.45 g
MgSO_4_	1.0 mM	0.45 g
KH_2_PO_4_	1.2 mM	0.16 g
MilliQ	N/A	To 1000 mL
**Total**	**N/A**	**1000 mL**

***Note:*** Aliquots of HEPES can be stored as 40 mL aliquots at −20°C for 1 year.

**Table:** HEPES3+ buffer

Reagent	Concentration	Amount
Preparation of HEPES3+ buffer		
Albuman (human serum albumin, stock 200 g/L)	5 g/L	400 μL
100 mM CaCl_2_ (in MilliQ-water)	1.0 mM	400 μL
2.5 M D-(+)-Glucose (in MilliQ-water)	5.0 mM	80 μL
HEPES-buffer (aliquot of 40 mL)	N/A	40 mL
**Total**	**N/A**	**±40 mL**

***Note:*** HEPES3+ should only be prepared before the experiment. It can be stored up to a week at 4°C but no longer. We recommend preparing fresh HEPES3+ each time before the start of a new experiment.

## Step-by-step method details

### Isolation of neutrophils and monocytes from whole blood using Ficoll layering

**Timing: neutrophil preparation 2 h**

**Timing: monocyte preparation 2 h + 3 h incubation**

All steps for the neutrophil and monocyte isolation are performed under BSL-1 conditions in a biosafety cabinet.

Here we describe how to isolate monocytes and neutrophils from whole blood samples (**Graphical abstract, step 1**). With this protocol, you can isolate monocytes and neutrophils with minimal activation of the cells and reduce cell death from handling procedures. This is important because activated neutrophils have a shorter lifespan than non-activated neutrophils.

***Note:*** This protocol yields ±2.0 × 10^6^–6.0 × 10^6^ neutrophils per mL blood and ±0.6–1.1 × 10^6^ monocytes per mL blood. In case of neutrophils, we recommend obtaining three 9 mL tubes for a full 96-wells plate and to seed 1.5 × 10^5^ cells per well. In case of monocytes, we recommend obtaining five 6 mL tubes for a full 96-well plate and seed 1.0 × 10^5^ cells per well. Check in advance how many cells you need for your experiment.

***Note:*** If desired, both monocytes and neutrophils can be obtained from the same donor for one high-throughput screening assay.

1. Transfer the blood from a sodium heparin blood tube and dilute with PBS2+ buffer at a 1:1 ratio.
  a. Before you begin, take two 50 mL tubes per blood donor and place them in a tube-rack in the biosafety cabinet.
  b. Fill one of the 50 mL tubes with 10 mL Ficoll Paque Plus.
  c. Check the blood volume while adding the blood to the empty 50 mL tube.
  d. Add the same volume of PBS2+ to the 50 mL tube containing blood in a 1:1 ratio.
    ***Note:*** A 9 mL blood tube usually contains 9 mL blood. Therefore, add 9 mL PBS2+ to 9 mL blood. This makes 18 mL total.
    **CRITICAL:** The 1:1 ratio is more important for successful Ficoll layering than the total blood volume. Always make sure this ratio is 1:1.
  e. Layer the 1:1 blood/PBS2+ mixture (so 18 mL) very slowly on top of the 10 mL Ficoll in the 50 ml tube.
    ***Note:*** Keep the 50 mL tube with Ficoll at a 30–45° angle from the worksurface, layer the 1:1 blood/PBS2+ on the Ficoll by putting the pipet straight at the wall of the tube and pipet out slowly.
    **CRITICAL:** You want to absolutely avoid mixing of the Ficoll and blood layers before centrifugation.
2. Centrifuge the Blood/PBS2+/Ficoll tube at 845 × *g* Relative Centrifugal Force (RCF) with acceleration at 2 and deceleration at 1 at 21°C for 20 min.
  a. Centrifuge the tube at low speed (acc. 2) and decelerate at the lowest break (dec. 1).
  b. Proceed to step 3 for monocyte isolation.
  c. Proceed to step 4 for neutrophil isolation.

**CRITICAL:** Centrifugation in step 2a, should always be performed at 21°C.

**CRITICAL:** After centrifugation, prevent disturbing the erythrocyte cell pellet since granulocytes are in the layer on top.

**CRITICAL:** The acceleration and deceleration are different per centrifuge model. Acceleration and deceleration of the Hettich Rotanta 460 ranges from 0 – 9. Where 9 is the maximum value and 0 is no break at all.

**Figure 1:**
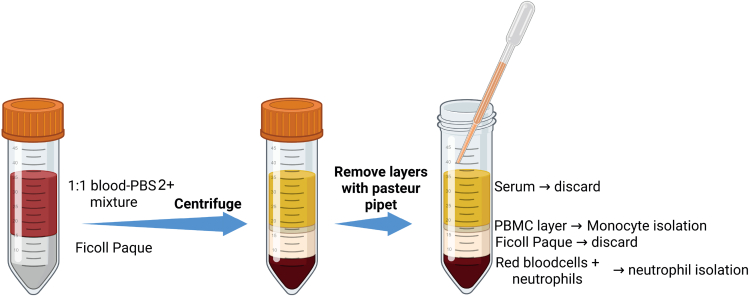
Schematic overview of Ficoll layering for different blood cell layers after centrifugation

***Note:*** For monocytes isolation, harvest peripheral blood mononuclear cells (PBMCs) by collecting the PBMC layer between the plasma layer and Ficoll layer as described in step 3. For neutrophil isolation, proceed from step 4. See Figure 1**,** for a schematic overview of the PBMCs and granulocytes layers after Ficoll layering and centrifugation as described in steps 1–2.

***Note:*** After step 2a, all solutions and cell suspension are kept at 4°C.

### Monocyte isolation

The monocytes are just below the plasma and Ficoll layer in the PBMC layer (Figure 1). In the steps described below, the protocol describes to obtain the PBMC layer, remove left over plasma and Ficoll with a Pasteur pipet and how to incubate the PBMCs for monocyte adherence.

**Figure 2:**
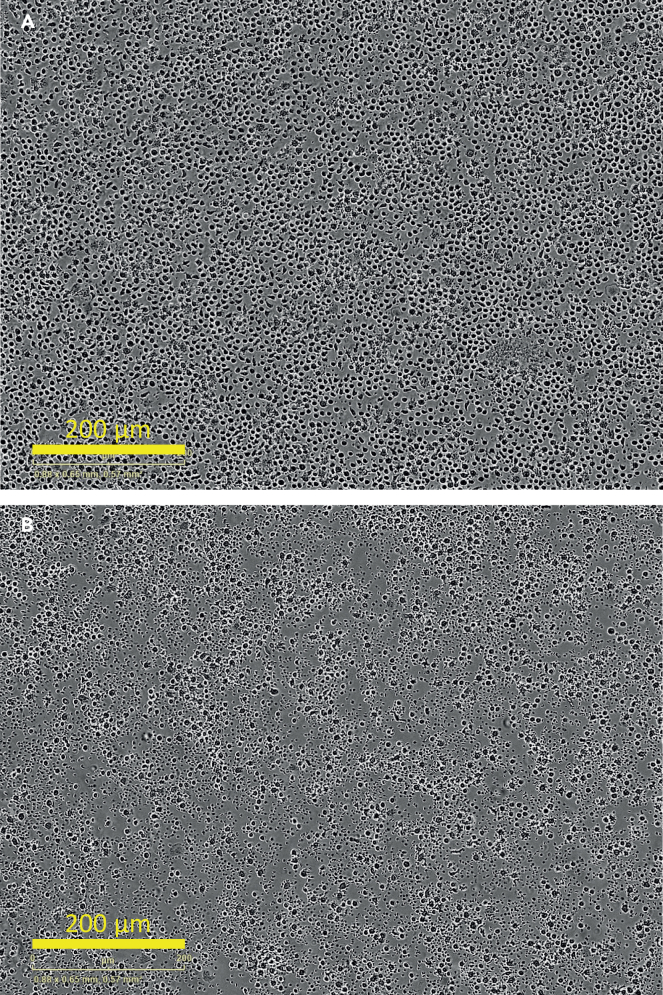
Representative phase-contrast image of plated PBMCs (A and B) PBMCs at start of 3-h incubation (A) and image of monocytes (control) after washing for removal of non-adherent PBMCs at time point 0 h (B). Magnification is 20× and scale bars represent 200 μm.

3. Carefully remove the PBMC layer and wash away left over plasma and Ficoll.
  a. In advance of the monocyte isolation, add 20 ml PBS2+ at 4°C to a new 50 ml tube.
  b. Carefully remove the PBMC layer with a Pasteur pipet found between the Ficoll and plasma layer. Try to avoid taking up too much plasma and Ficoll.
  c. Transfer the PBMCs with the Pasteur Pipet to the tube containing 20 mL PBS2+.
  d. Centrifuge the cells at 475 × *g* at 4°C for 5 min and wash again in 10 mL PBS2+ buffer.
  e. Resuspend the PMBC pellet in RPMI 1640 containing, 2 mM L-glutamine and 10% human pooled serum. See the preparation of supplemented RPMI 1640 in steps 16a-d in the before you begin section.
  f. Count the cells and seed 5 × 10^5^ of the PBMCs for and estimated 1 × 10^5^ monocytes per well in a black/clear polystyrene flat bottom (Corning/Falcon) imaging 96-well plate.
    **CRITICAL:** You cannot precisely count the number of monocytes in this protocol.
    ***Note:*** The number of monocytes in the PBMC compartment is estimated to be ±20%. Therefore, we recommend dividing the total cell count of the PMBC layer by 5 for the monocyte count.
  g. Let the monocytes adhere to the bottom of the imaging plate at 37°C for 3 h.
    ***Note:*** In the time of the monocyte incubation to the plate, you can proceed to the neutrophil isolation described in step 4.
  h. After 3-h incubation, remove the incubation medium with a vacuum suction device in the flow cabinet.
  i. Add supplemented RPMI 1640, containing 1% HPS with a multi-channel pipet.
  j. Wash the plate with RPMI 1640 supplemented with 1% HPS and remove with the multi-channel pipet.
  k. If you want to isolate neutrophils, proceed to step 4. If you want to skip this and want to prepare the particles, proceed to step 7 under particle preparation.
    ***Note:*** See Figure 2 to see how correct plating of PBMCs and monocytes after 3-h incubation and washing should look like.
    ***Note:*** After incubation, only monocytes will adhere to the plate. Other cells are washed away.
    **CRITICAL:** Never add RPMI 1640 supplemented with 1% human pooled serum directly on top of the cells but place the pipet in an angle adjacent to the wall.
    **CRITICAL:** Do not remove the medium of all the conditions at once for the whole plate to prevent prolonged air exposure and drying out of the cells.

### Neutrophil isolation

The neutrophils are in the granulocyte layer directly above the erythrocyte pellet (Figure 1). All the plasma, PBMCs and Ficoll above the granulocytes and erythrocytes should be removed if you want to isolate neutrophils in your experiment. Remove the remaining Ficoll and plasma including the PBMC layer (if not collected for monocytes) with a Pasteur pipet.

**CRITICAL:** It is important to start neutrophil isolation as soon as possible after blood draw. Neutrophils become more activated by remaining in the blood collection tube, which reduces viability. Therefore, it is critical to start with the experiment as soon as blood collection is performed and start isolation within 1 h after blood collection.

**CRITICAL:** For removal of all the other layers except the neutrophils and erythrocytes, it is essential that the erythrocyte and granulocyte layer are not disturbed. Otherwise, this will lead to reduced neutrophil numbers and more contamination with other cells.

4. Remove all the plasma, PBMCs and Ficoll with a Pasteur pipet.

***Note:*** You can discard plasma, PBMCs and Ficoll. If you want to use monocytes for experimental testing, perform step 3 for monocyte isolation as described above.

5. Use a sterile cotton swab to clean the inner sides of the 50 mL tube.

**CRITICAL:** If you want to study the effects of MNPs on neutrophils, you want to get rid of all other cell types in the PMBC layer. Therefore, you should swab the wall of the tube to get rid of the ‘sticky’ PMBCs as best as possible.

**Figure 3:**
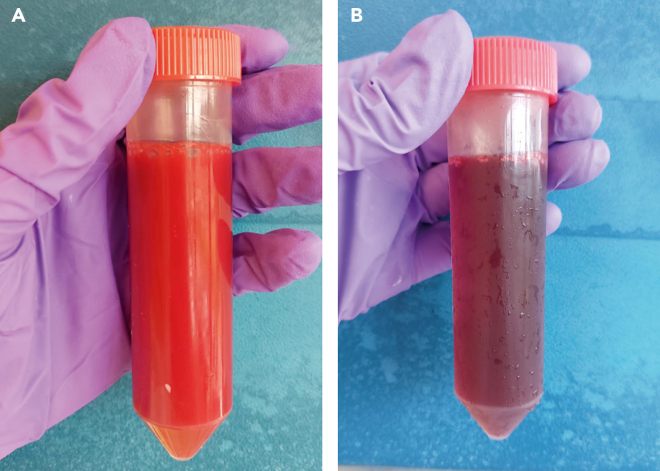
Erythrocyte lysis by addition of ACK-lysis buffer (A and B) Representative images of unshocked (A) and lysed (B) human blood after Ficoll layering steps.

6. Resuspend the remaining erythrocyte and granulocyte pellet in ACK-lysis buffer.
  a. Add 35 mL ACK-lysis buffer to the ±5 mL granulocyte/erythrocyte pellet in the 50 mL tube. You need a ratio of 7:1 of ACK-lysis:cell volume.
  b. Incubate the cells in ACK-lysis buffer at 4°C for 15 min.
  c. Shake the tube a few times after ±7 min during the 15 min incubation time, see Figure 3.
    ***Note:*** The solution should change from a turbid and light red color to a more transparent and deep red color. 15 min is the average time for the shift/erythrocyte lysis to occur, but time may vary depending on the blood donor.
  d. Centrifuge at 475 × *g* 4°C for 5 min
  e. Aspirate the supernatant with a vacuum suction system.
  f. Perform a second lysis by resuspending the pellet in 25 mL ACK-lysis buffer.
  g. Centrifuge the resuspended pellet immediately at 475 × *g* at 4°C for 5 min.
  h. Aspirate the supernatant with a vacuum suction system.
  i. Wash the cells by resuspending the pellet in 10 mL PBS2+ at 4°C.
  j. Centrifuge immediately at 475 × *g* at 4°C for 5 min.
  k. Resuspend the cell pellet in 500 μL of HEPES3+ buffer and count the granulocytes.
    ***Note:*** In this protocol, we counted the neutrophils with a Cell Dyn Emerald (Abbott). The Cell Dyn Emerald gives you a percentage of granulocytes of the total white blood cells in the sample. For neutrophil isolation, it shows you remaining ‘contamination’ with PBMCs, erythrocytes or platelets. The granulocyte compartment also contains eosinophils, but these make up less than 5% of the total leukocytes in blood and are therefore neglected.
    **CRITICAL:** If the steps of the Ficoll layering are followed correctly, neutrophil purity will be minimally 90%.
7. Adjust the count of the whole blood count for the percentage of granulocytes (±90%) and dilute the granulocytes to 17.5 × 10^6^ cells/mL and store at 4°C until for later use.

**CRITICAL:** Do not use neutrophil purity counts of less than 85%.

**CRITICAL:** Use the neutrophils within 2 h of isolation.

### Particle preparation by opsonization in human pooled serum

**Timing: 30 min, performed under sterile conditions in a BSL-1 biosafety cabinet**

In our experience with particle toxicity testing, we found that opsonizing MNP particles with human pooled serum (HPS) is necessary for improved particle engulfment.^1^ Therefore, the particles are incubated in HPS prior of the high-throughput cell death screening in neutrophils and monocytes. Here, we describe the steps needed for particle opsonization.

***Note:*** The opsonization in step 7 can be done concurrently during the waiting steps of neutrophil isolation or the monocyte incubation steps.

***Note:*** HPS can be heat-inactivated at 56°C for 30 min or substituted with plasma or serum from the same blood donor if the complement system interferes with answering your research question.

***Note:*** For different cell types and different particles or compounds this opsonization step might not be necessary.

8. All 0.20 μm, 1 μm and 10 μm polystyrene and 1 μm and 10 μm silica beads are opsonized by pretreatment in human pooled serum (HPS) prior to further dilution in cell medium.
  a. Dilute the 2.7% w/v 0.20 μm polystyrene particle stock 1:27 in HPS by adding 7.4 μl particle stock to 192 μL HPS.
  b. Dilute the 10% w/v 1 μm polystyrene particle stock 1:100 in HPS by adding 2 μl to 198 μl HPS.
  c. Dilute the 10% w/v 10 μm polystyrene particle stock 1:100 in HPS by adding 2 μl to 198 μl HPS.
  d. Dilute the 5% w/v 1 μm silica particle stock 1:50 in HPS by adding 4 μL particle stock to 196 μL HPS.
  e. Dilute the 5% w/v 10 μm silica particle stock 1:50 in HPS by adding 4 μL particle stock to 196 μL HPS.
  f. Incubate all the particles in HPS for 30 min at 21 ° C.

***Note:*** The particles in this protocol have a different stock concentration, so a different amount of stock concentration is required to expose the cells to the same weight-based concentration.

***Note:*** Although the stock concentrations of the particles are different, we assume differences in HPS volume are negligible because you add additional HPS to the end mixture.

### Adding neutrophils and particles to the 96-well plate

**Timing: 45 min, performed under sterile conditions in a BSL-1 biosafety cabinet**

The neutrophils and particles are combined in a 1.5 mL Eppendorf tube before they are added to the compatible imaging plate (**Graphical abstract step 2**).

**CRITICAL:** Combine all the components in the Eppendorf tube prior to adding the neutrophils.

9. During the incubation of the particles in the HPS, prepare and label the Eppendorf tubes.
10. Prepare 440 μL of the neutrophils in compound mixture according to the steps and sequence as described in Table 1. Step by step addition of compounds neutrophil screening.
  a. Add 366.44 μL HEPES3+ buffer to an Eppendorf.
  b. Add 4.4 μL of a 100 mM CaCl_2_ solution in MilliQ-water to the Eppendorf.
  c. Add 4.4 μL 1:1000 f-MLF diluted in HEPES3+ buffer to the Eppendorf.
    i. Predilute the f-MLF 1:1000 in HEPES3+ buffer
  d. Add 1.46 μL AnnexinV-FITC to the Eppendorf.
  e. Add 0.44 μL SytoxAADvanced to the Eppendorf.
  f. Add the opsonized particles in HPS in the concentration as mentioned in Table 1 to the Eppendorf.
  g. Add additional HPS to obtain the total HPS 10% as described in Table 1.
  h. Combine 18.86 μL neutrophil cell suspension with the compound mix in the Eppendorf.
  i. Mix the compound mix by pipetting up and down.
    **CRITICAL:** Add the cells last to limit interaction of cells with the particles prior imaging. The other components do not need to be in a specific order. We recommend pipetting as quickly as possible after homogenization by pipetting up and down.
  j. Add 200 μL of the solution from the Eppendorf into the 96-wells imaging plate.
  k. Each condition consists of two wells containing 200 μL in the 96-wells plate.
    **CRITICAL:** Neutrophil activation can be heterogenous between donor individuals based on genetic background, time of the day or pathogenic infections. Adding N-Formyl-L-methionyl-L-leucyl-L-phenylalanine (f-MLF) to a concentration of 10^−9^ M to ensure all neutrophils reach a maximum level of activation so that the response to particles is the same for each donor.
    **CRITICAL:** All conditions mixtures should contain 10% HPS in the end, see Table 1. For the control condition without pretreated particles, 44 μL HPS should be added to a 440 μL volume.
    ***Note:*** Prepare a total of 440 μL solution per duplicate, this allows for a 10% pipetting error.
    ***Note:*** For the plastic and silica concentrations, the HPS will not be completely 10% in the end, but lower since the particles are pretreated in HPS. The PS 1 μm PS and 10 μm are pretreated 1:100 in HPS, the PS 0.20 μm PS 1:27 (stock is 2,7% w/v or 27000 mg/mL), and the silica 1 μm and 10 μm silica 1:50. However, we consider this slightly lower HPS concentration negligible.
    ***Note:*** AnnexinV-FITC is added at a final concentration of 0.3 μg/mL. This is optimized based on titration before and the same concentration used by Giulio et al.^1^ The SytoxAADvanced concentration of 1 μM is based on the manufacturer recommendations. If the stains do not work, titrate the dyes to make them optimal.

**Table 1:** Step by step addition of compounds neutrophil screening

Steps	Reagent	Final concentration	Amount
1.	HEPES3+	N/A	366.44 μL
2.	CaCl_2_ 100 mM	2 mM (1mM from original HEPES3+ and additional CaCl_2_ mentioned here)	4.4 μL
3.	f-MLF (prediluted in HEPES3+ 1:1000 to μM from 1 mM in DMSO)	10^−9^ M	4.4 μL
4.	AnnexinV-FITC 90 μg/mL	0.3 μg/mL	1.46 μL
5.	SytoxAADvanced 1 mM in DMSO	1 μM	0.44 μL
6.	Polystyrene or silica particles opsonized in HPS	0 μg/mL (control)	0 μL
		1 μg/mL	0.44 μL
		10 μg/mL	4.4 μL
		100 μg/mL	44 μL
7.	Additional human pooled serum	10% for 0 μg/mL (control)	44 μL
		±10% for 1 μg/mL	43.56 μL
		±10% for 10 μg/mL	39.6 μL
		±10% for 100 μg/mL	0 μL
8.	Neutrophils in HEPES3+ (17.5 × 10^6^ cells/mL = ±330000 cells in 18.86 μL	7.5 × 10^5^ cells/mL (= equivalent to 1.5 × 10^5^ cells/200 μL	18.86 μL
	**Total**	**N/A**	

### Preparation of particle mixture in the 96-well plate for adherent monocytes

**Timing: 45 min, performed under sterile conditions in a BSL-1 biosafety cabinet**

Monocytes need to be added to the well in supplemented RPMI 1640 with 2 mM L-glutamine and 10% HPS prior to the addition of particles. The monocytes are found in the PBMC layer and they need to attach to the plate prior particle exposure, see steps 3a-j for monocyte isolation. Therefore, you need to prepare the compound mix separately from the monocytes as described below.

11. During the incubation of monocytes and the opsonization of the particles in the HPS, prepare and label the Eppendorf tubes for the different conditions.
12. Prepare 440 μL of the compound mixture for monocytes according to the steps and sequence as described in Table 2. Step by step addition of compounds monocyte screening.
  a. Add 389.7 μL RPMI supplemented with 2 mM L-glutamine buffer to an Eppendorf.
  b. Add 4.4 μL of a 100 mM CaCl_2_ solution in MilliQ-water to the Eppendorf.
  c. Add 1.46 μL AnnexinV-FITC to the Eppendorf.
  d. Add 0.44 μL SytoxAADvanced to the Eppendorf.
  e. Add the opsonized particles as described in Table 5 in HPS to the Eppendorf.
  f. Add additional HPS to have 10% as described in Table 2 to the Eppendorf.
  g. Mix the compound mix by pipetting up and down.
  h. Remove the RPMI 1640 containing 1% HPS wash buffer just before addition of the compound solution as described in step 3 h.
  i. Add 200 μL of the compound solution from the Eppendorf to the imaging plate.
  j. Each condition consists of two wells containing 200 μL in the 96-wells plate.

**CRITICAL:** You should use RPMI 1640 supplemented with 2 mM L-glutamine without HPS to prepare the compound mix. Otherwise, the final HPS concentration exceeds 10% in the end. The HPS is added separately to make sure this is 10% of the total volume.

**CRITICAL:** It is important to tilt the plate to prevent disturbance of the cell layer while pipetting the compound mix.

***Note:*** Monocytes do not need activation by f-MLF. Therefore, this is not added to the monocyte compound mix.

***Note:*** Prepare a total of 440 μL solution per duplicate, this allows for a 10% pipetting error.

**Table 2:** Step by step addition of compounds monocyte screening

Steps	Reagent	Final concentration	Amount
1.	RPMI 1640, supplemented with 2 mM L-glutamine	N/A	389.7 μL
2.	CaCl_2_ 100 mM	1 mM	4.4 μL
3.	AnnexinV-FITC 90 μg/mL	0.3 μg/mL	1.46 μL
4.	SytoxAADvanced 1 mM in DMSO	1 μM	0.44 μL
5.	Polystyrene or silica particles opsonized in HPS	0 μg/mL (control)	0 μL
		1 μg/mL	0.44 μL
		10 μg/mL	4.4 μL
		100 μg/mL	44 μL
6.	Additional HPS	10% for 0 μg/mL (control)	44 μL
		±10% for 1 μg/mL	43.56 μL
		±10% for 10 μg/mL	39.6 μL
		±10% for 100 μg/mL	0 μL
	**Total**	**N/A**	**440 μL**

### Setting up the Incucyte S3 instrument for visualization of cell death over time

**Timing: 15–30 min**

After adding the conditions to the imaging plate, you can add the plate to the Incucyte S3 Live Cell Imaging System to measure cell death over time **(Graphical abstract, step 3)**. Here, the protocol describes the steps to place the plate in the Incucyte S3 and to adjust the software settings to measure over time the phase-contrast, the green- and the red-signal with your preferred time intervals.

13. Place the imaging plate with your experimental conditions plate in the Incucyte for visualization over time.
  a. Place the imaging plate in the Incucyte instrument and press ‘+’ in the Schedule overview of the software to start a new measurement.
    **CRITICAL:** It is important that you place the 96-wells plate correctly in the plate holder and not tilted. Otherwise, the whole or part of the plate will not be in focus. The plate should not move when placed in the plate holder.
  b. In the tab ‘Scan repeatedly or ‘Scan Once’, select ‘Scan on Schedule’.
  c. Press ‘Next’ to start a new measurement.
  d. In the tab ‘Scan Type’, choose ‘Standard’.
  e. Press ‘Next’, to go to ‘Scan Settings.
  f. In the ‘Scan Settings’, check the box for Cell-by-Cell options for ‘None’.
  g. Check the boxes for ‘Phase’, ‘Green’ or ‘Red’ for the imaging channels you want to measure.
  h. Change the objective to a 20× magnification.
  i. Press ‘Next’ to go to the next tab for selection of the vessel.
  j. In the tab ‘Vessel Selection’, select the serial number of the plate that you use for imaging.
    ***Note:*** In this high-throughput screening assay, we use ‘Phase’ to observe the cells and presence of particles, ‘Green’ to visualize the AnnexinV-FITC as membrane cell death marker and ‘Red’ to visualize SytoxAADvanced as DNA-bound cell death marker.
    ***Note:*** The default setting for the acquisition time (ms) for the Green Channel is 300 ms and 400 ms for the Red Channel. In our experience, the default worked well and did not require further optimization.
    ***Note:*** The acquisition time is dependent on the intensity of your dye. Adjust the acquisition settings or titrate the dye in advance for optimal imaging.
    ***Note:*** The 20× objective does not visualize the entire well.
  k. Select the imaging plate you use in the plate overview and press ‘Next’ to go to the next tab.
  l. In the tab ‘Channel Location’ select the vessel in the plate drawer where your plate is located.
  m. Press ‘next to go to the next tab.
  n. In the tab ‘Scan Pattern’ you select the wells you want to image and the number of images you want to make per well.
  o. Press ‘Next’ to go to the next tab.
  p. Select 3 images per well and proceed to the ‘Vessel Notebook’ in the next tab.
    ***Note:*** In our high-throughput screening assay, we use the polystyrene flat bottom imaging 96-well plate (Corning/Falcon); Cat#353219).
    **CRITICAL:** In this protocol, each biological condition is measured as the mean of two wells, based on three images for each well. The number of images you take per well and number of conditions influence the scanning time per interval. More images per well result in longer scanning times. The Incucyte can only image 30 min per hour to prevent overheating. Make sure that the intervals and the scanning time in total (so including other plates in the Incucyte) do not exceed 30 min per hour.
14. In the tab ‘Vessel Notebook’ you can name your vessel and indicate the cells used in the experiment.
  a. You can also create (press ‘+’) or import a plate map (press folder) to know which conditions you have in the plate.
  b. Press ‘Next’ when you are finished with the plate map to go to the next tab.

**Figure 4:**
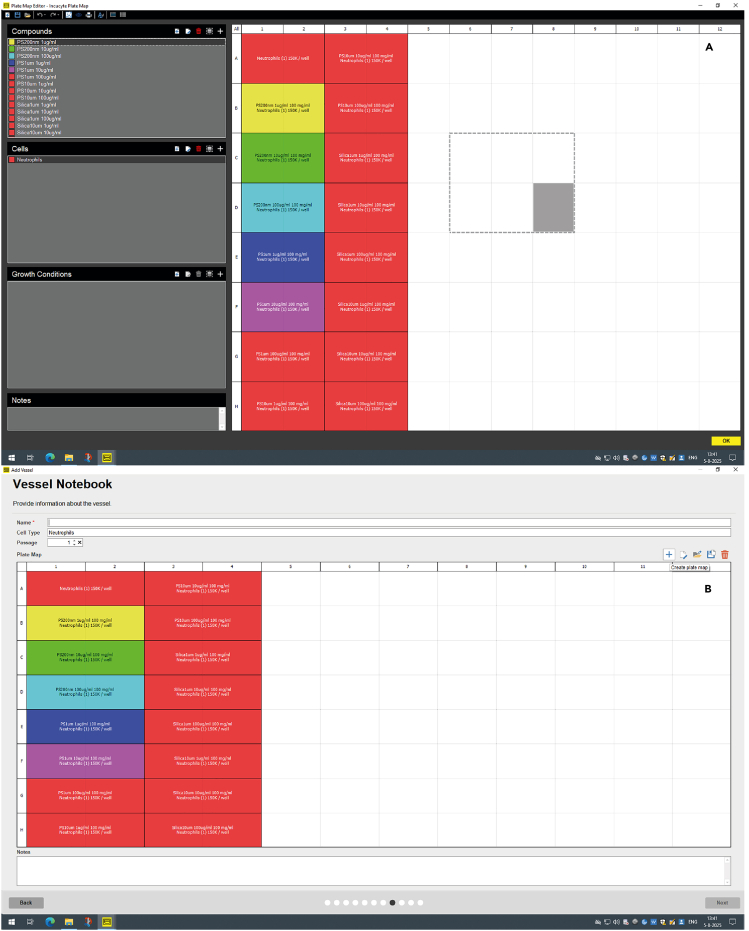
Set up of the plate layout in the Incucyte S3 analysis software Overview in the Incucyte S3 analysis software where you can adjust the plate layout (A) and the Vessel Notebook (B).

***Note:*** See Figure 4 for the plate map overview we used in this experiment.

***Optional:*** Although not required, we strongly recommend making a plate map if there are multiple different conditions. The plate map is necessary for the export of data in later steps if you want to have the data clustered per condition. The plate map information is needed to cluster the analysis in the Incucyte S3 software and for analyzing the difference between conditions. Otherwise, you have to do this manually. If you do not prepare a plate, you can only cluster the analysis in whole ‘rows’ or ‘columns’.

***Optional:*** You can make the plate map as detailed as you want with information about the conditions, cells and concentrations.

***Optional:*** Once you have prepared a plate map with all conditions and locations on the plate, you can save and use it for the next experiment. For a repeat of the experiment, you can start with a new plate map or adjust and add new conditions to the already saved plate map used before.

15. In the tab ‘Analysis Setup’ keep the ‘Analysis Type’ on default ‘<Defer Analysis until later>’.
  a. Press ‘Next’ to proceed to the next tab ‘Scan Schedule’.

**CRITICAL:** We recommend to start scanning at least 15 min after placing the plate in the Incucyte vessel holder. The delayed start time at a minimum of 15 min is to make sure that any condensation has dissipated from the plate which may interfere with the automatic focusing of the Incucyte.

**Figure 5:**
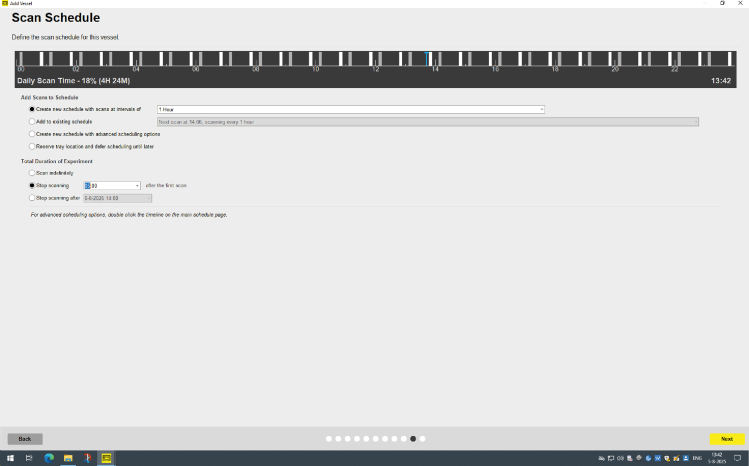
Overview of the Scan Schedule tab of the Incucyte S3 Software

16. In the tab ‘Scan Schedule’ check the box for ‘Create new Schedule with Scans at Intervals’ of any other interval you want to scan.
  a. In the ‘Scan Schedule’ tab, select the intervals of your preference.
  b. You can click and drag the white column of the Interval in the visualized time schedule at the top of the window and modify when you want to start your measurement, see Figure 5.

**CRITICAL:** Check that your experiment does not interfere with scanning intervals of other independent experiments and make sure that the intervals and the total scanning time of the whole Incucyte S3 in total do not exceed 30 min per hour. If this is the case, adjust your intervals, images per scan, or which channels to measure to reduce imaging time until the software gives no warning that the 30 min per hour threshold is passed.

***Note:*** The number of intervals and when to start your experiment is of your own preference, but usually when all the conditions are set you want to start imaging as quickly as possible.

17. Under the tab ‘Total Duration of the Experiment’ select the box ‘Total Duration of the Experiment’.
  a. Check ‘Scan Indefinitely’ or set at the time that you want in ‘Stop Scanning after the first scan’.
    ***Note:*** In the ‘Scan Schedule tab’ we prefer to stop scanning after 5 days. Longer scanning is not needed for toxicity screening in neutrophils and monocytes.
  b. Press ‘Next’ to go to the next and last tab ‘Summary’.
18. The tab ‘Summary’ summarizes the settings of the experiment.
  a. Read this through to see if all settings are correct or need adjustment.
  b. If everything is correct, press ‘Add to Schedule’.
19. After the Incucyte S3 has made the first scan, check regularly if the imaging plate remains completely in focus.

***Note:*** Although we use 1-h intervals in this experimental set-up. With multiple imaging plates in the Incucyte S3, 2- up to 4-h intervals will also provide you with the same results. However, sometimes the Incucyte S3 cannot find focus. If this happens in the first time points in case of a 2- or 4- h scanning interval, you cannot determine the initial slope anymore.

**CRITICAL:** If plate remains out of focus, see how you can solve this problem in the troubleshooting section.

### Addition of 0.5% Triton X-100 in PBS to achieve maximum cell death

**Timing: 15 min, performed under sterile conditions in a BSL-1 biosafety cabinet**

In this section, the protocol describes to add 0.5% Triton X-100 to the wells after at least 72 h of imaging to achieve maximum cell death in the wells of each experimental condition. See step 17 before you begin for preparation of 0.5% Triton X-100 in PBS.

**Figure 6:**
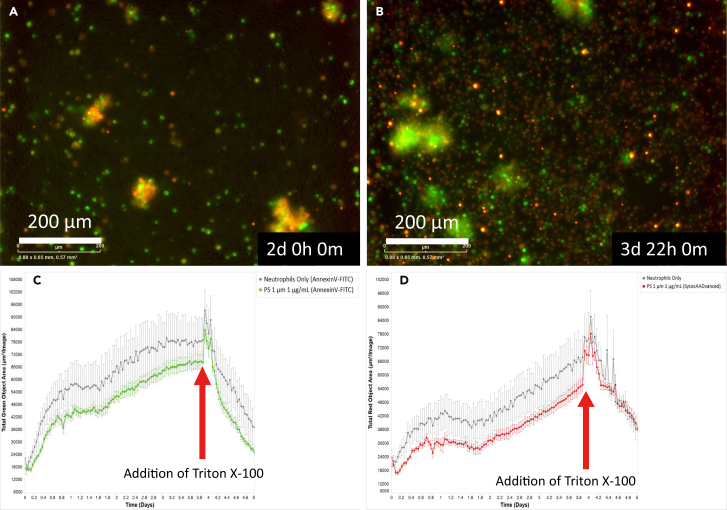
Triton X-100 effects on neutrophil survival of one representative experiment (A–D) Images of neutrophil control before (A) and after (B) 0.5% Triton-X-100 addition at time point 94 h. Kinetic survival data of non-normalized neutrophil survival graphs for control (gray line) and 1 μg/mL dose of 1 micrometer (μm) polystyrene (PS) (green line) for AnnexinV-FITC signal (C) and control (gray line) and 1 μg/mL dose of 1 μm PS (red line) for SytoxAADvanced signal (D). The red arrows point out when 0.5% Triton X-100 is added at 93 h after incubation. The maximum cell death for the AnnexinV curves is reached after 94 h and for the SytoxAADvanced curve after 97 h. The survival graphs are based on the average of two 96-wells of 3 images of one experiment, including standard error, exported from the Incucyte analysis Software. Scale bars represent 200 μm.

20. Open your experiment in the ‘View’ tab in the software.
  a. Check that the plate is imaged for a minimum of 72 h.
    **CRITICAL:** Make sure that you add the 0.5% Triton X-100 in between intervals when the Incucyte is not scanning the plate.
  b. Remove the imaging plate from the Incucyte S3 and place it in a biosafety cabinet under BSL-1 conditions.
  c. Add 20 μL 0.5% Triton X-100 in PBS to all wells to induce maximum cell death in each well and condition.
    **CRITICAL:** During addition of the 0.5% Triton X-100, add the solution against the wall of the well and do not resuspend to avoid disturbance in the field of view. Otherwise, cells may be washed away to one side of the well which will also affect the result.
    ***Note:*** We recommend to add 0.5% Triton X-100 right after a scan to make sure condensation from moving the plate in or out the Incucyte incubator, dissipates before the next scan. Otherwise, the next scan might not be in focus.
  d. Check regularly during the subsequent intervals to see when all cells reach maximum cell death.
    **CRITICAL:** Image for a total of 5 days where after at least 72 h of incubation the 0.5% Triton X-100 is added to make sure the cells have enough time to permeabilize and reach maximum cell death.
    ***Note:*** Representative examples of the addition of Triton X-100 to neutrophil control and 1 μg/mL of 1 μm PS can be found in Figure 6. In this example, 0.5% Triton X-100 is added after 93 h but the peak of 100% cell death is reached after approximately 94 h for AnnexinV-FITC signal and 97 h for the SytoxAADvanced signal.
    ***Note:*** You do not need to reach total 100% in the immediate next scan, but you can check regularly via the Incucyte Software when a 100% cell death occurs. This can vary from a couple of hours to a whole day. If the cell death signal decreases after 0.5% Triton X-100 addition, it means that the maximum cell death has already occurred in earlier scans.
    ***Note:*** It is possible that signal from AnnexinV-FITC and SytoxAADvanced signal decreases after the maximum is reached because of complete permeabilization and disintegration of the cells by the 0.5% Triton X-100.
21. After 5 days you can remove the imaging plate and discard it.

### Setting the mask for analysis of cell survival in the Incucyte S3 software

**Timing: 15–30 min setup of analysis masks**

**Timing: 3–4 h analysis run time**

Masking of the cell death markers is an important step in the high-throughput screening analysis **(Graphical abstract, step 4).** Here in steps 22–31, the protocol describes how to correctly mask for the fluorescent signal of the area in the Incucyte S3 software and export it to a text file.

**CRITICAL:** If one condition or a whole experiment needs drastically different analysis settings than other experiments, you can consider excluding it.

**CRITICAL:** We recommend keeping the threshold values for masking similar between experiments as this helps for objective analysis. However, one should always consider if the masking threshold is correctly applied.

***Note:*** Setting the perfect mask will be difficult to achieve, so go for the best fit that suits all conditions in one experiment.

***Note:*** Setting the mask is partially subjective, since the Incucyte Software shows a suggestion that you can fine-tune by increasing or decreasing the threshold values.

22. To set the mask, open your experiment in the Incucyte Software and go to ‘View’.
  a. Press ‘Launch Analysis’.
  b. In the pop-up tab ‘No Spectral’, select ‘Yes’ to go the next tab.
  c. In the next tab ‘New or Use Existing Analysis Definition’ press ‘+ Create New Analysis Definition’.
  d. Press ‘Next’ to go to the new tab for analysis.
  e. In the tab ‘Analysis Type’, select ‘Basic Analyzer’ and proceed to ‘Next’ to go to next tab.
  f. In the tab ‘Image Channels’, select the channels you want to analyze.
  g. Press ‘Next’ to go to the ‘Image Set Selection’ tab.
23. In the tab ‘Image Set Selection’ you go to a representative time point that shows both positive and negative cells, for example a 24-h time point.
  a. In the overview of the 96-wells plate you select representative images/wells of each condition to base your masking on.
  b. When finished, press ‘next’ to go to the new ‘Analysis Definition’ tab.

***Note:*** You can select as many representative images as possible. With more images it will take more time to check the masking on all of them, but it will be more representative. One of each condition at the 24-h time point is usually sufficient.

**Figure 7:**
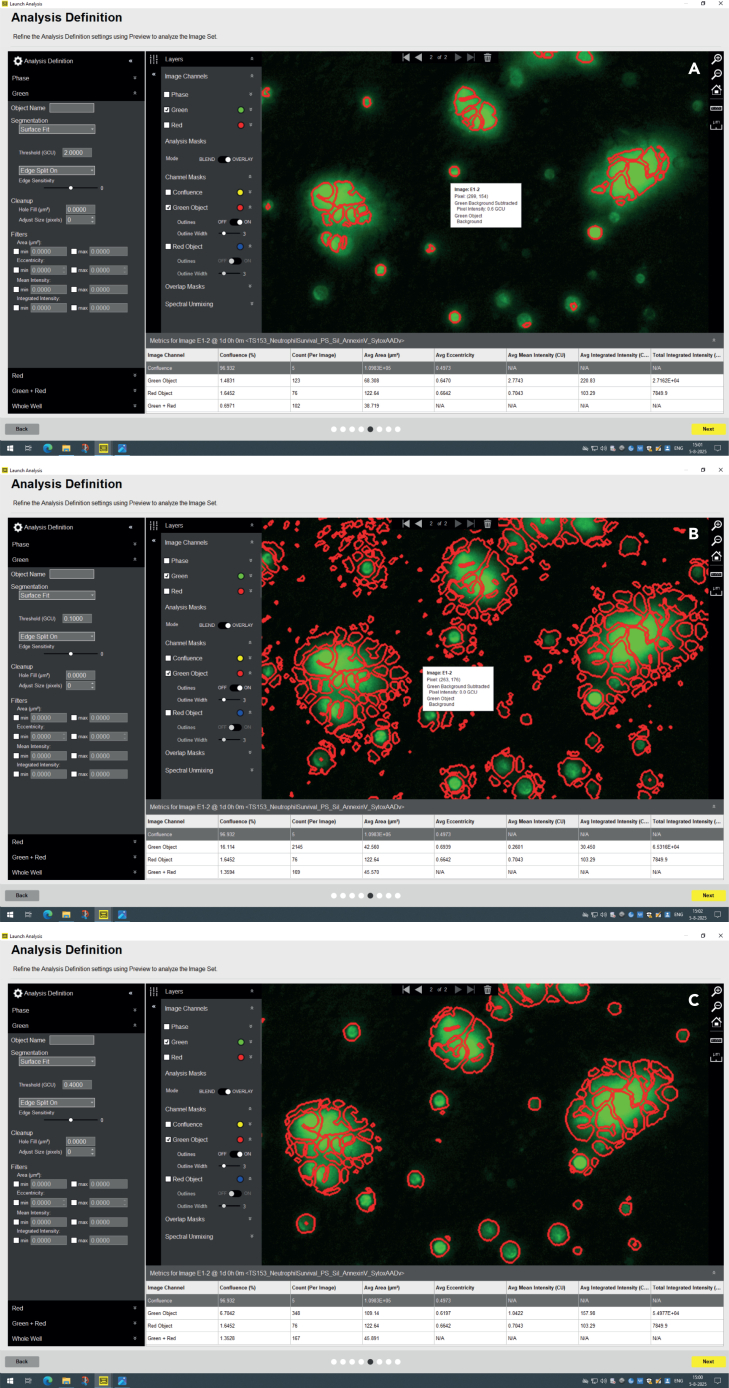
Overview of the manual masking in the Incucyte S3 Analysis Software (A and B) Masking for AnnexinV-FITC signal (Green Channel) for well E1 (PS 1 μm 100 μg/mL condition) for time point 24 h. The different masking is visualized a check under Green Object (lining in Red). Under masking of the AnnexinV with threshold at 2.000 (default) (A), over masking with threshold at 0.1000 (B) and correct masking with threshold at 0.4000 (C).

24. In the tab ‘Analysis Definition’ under ‘Green Channel’ tab, adjust the Threshold (GCU) based on every selected image to make sure that the mask is optimal for all conditions.
  ***Note:*** We consider the mask optimal when as little background as possible is taken along for the masking and that also the masking truly reflects the cell death signal in the field of view. See Figure 7 for correct and incorrect masking with over or under estimation of the signal and their settings.
  a. Press ‘Preview Current’ to check if the mask is correctly applied for the green channel (AnnexinV-FITC in this protocol) and change if this needs adjustment.
    ***Note:*** In the experimental set up of this protocol, we changed the Threshold (GCU) from 2.000 (default) to 0.4000. This depends on the background per imaging plate and per experiment and conditions but should be similar.
  b. Make sure the masking area is the same within one experiment and has similar results between different experiments with same conditions.
    **CRITICAL:** You have to critically assess the threshold settings for each experiment, and you are allowed to adjust the threshold settings of the green and red channel. However, you can also adjust the threshold by adjusting Segmentation, Edge split on or off, Clean up or Filters. If you use these settings, do this also for the next experiments.
    **CRITICAL:** It is essential that the masking of background is minimized so that the mask truly reflects cell death signal in the field of view.
25. Repeat the previous step for the ‘Red Channel’ (SytoxAADvanced in this protocol) tab.
  a. After the mask is set, press ‘Next’ to go to the next ‘Scan Time and Wells’ tab.
26. In the tab ‘Scan Time and Wells’, select all the wells and time points you want to include in your analysis.
  ***Note:*** In the high-throughput screening assay of this protocol, we included all time points up to 5 days including the time points after addition of 0.5% Triton X-100 to make sure that we reached the maximum value to do the normalization.
  a. Press ‘Next’ to go to the next ‘Save and apply Analysis Definition’ tab.
27. In the tab ‘Save and apply Analysis Definition’, give the analysis a name.
  a. Press ‘Next’ to go to the last ‘Summary’ tab.
28. In the ‘Summary’ tab, check the settings before launching the analysis.
  a. Press ‘Next’ when everything is set correctly.
  b. The analysis will now run.

***Note:*** The time for the analysis to run depends on the number of conditions, channels and time intervals. The screening described in this protocol takes 3–4 h.

29. When the analysis has completed, you export the data by clicking on ‘Graph Metrics’.
  a. Select under the Green or Red subtab ‘Total Area (μm^2^/Image)’.
  b. Select all time points up to the moment that the maximal value is reached (this might be a couple of hours after addition of 0.5% Triton X-100).
  c. Select all the wells that you want to export.
    ***Note:*** In this protocol, we select ‘Plate Map Replicates’ in Select Grouping, to make sure that all conditions have the name that you assigned to each condition during setting up of the Plate Map.
  d. Press ‘Export Data’.
  e. In the tab ‘Graphing Export’, select where you want to save your file and check the box ‘Include Experiment details in header’.
  f. Press ‘Export’ to export the file as text file in the selected folder.
  g. The data of the green and red channel are exported as separate text files.
    ***Optional:*** You can also export the data to ‘clipboard’ and paste directly in a text or excel file.
    ***Note:*** Here, we export the data via the ‘Export Data’ because we want to directly have the data in a text file to manually change the decimal ‘.’ to ‘,’. You can also change to copy it to clipboard into Excel, but then you must change the R script for normalization.
    ***Note:*** In this protocol, each experiment consists of conditions of two technical replicates with three images each per well (so six images per condition per experiment). The protocol describes exporting the outcome of the mean total area of one condition in one experiment, based on the mean of six images by the Incucyte Analysis Software.
    ***Optional:*** You can change this by exporting the data of each well separately.
30. To do this, ‘Select Grouping’.
31. Select ‘None’ and export data under ‘Layout’ as: ‘Row by Row A1, A2, A3, … B1, B2, B3, …’ and under ‘Other Options’: ‘Break Data down in individual images’.

***Note:*** This protocol uses the Total Area **(**μm^2^/Image) instead of the object count since clusters of death cells may be quantified as one object while it consists of multiple death cells. Therefore, we recommend Total Area as better measurement for quantifying total cell death compared to cell count.

## Expected outcomes

This protocol describes how neutrophil and monocyte cell death can be determined from the AnnexinV-FITC or SytoxAADvanced signal of the area of death cells, followed over time. Neutrophil and monocyte cell death follows similar kinetics for both cell death stains after particle exposure. If you expect apoptotic cell death, the extracellular phosphatidyl serine stained by AnnexinV-FITC precedes cell permeability and subsequent DNA staining by SytoxAADvanced.

**Figure 8:**
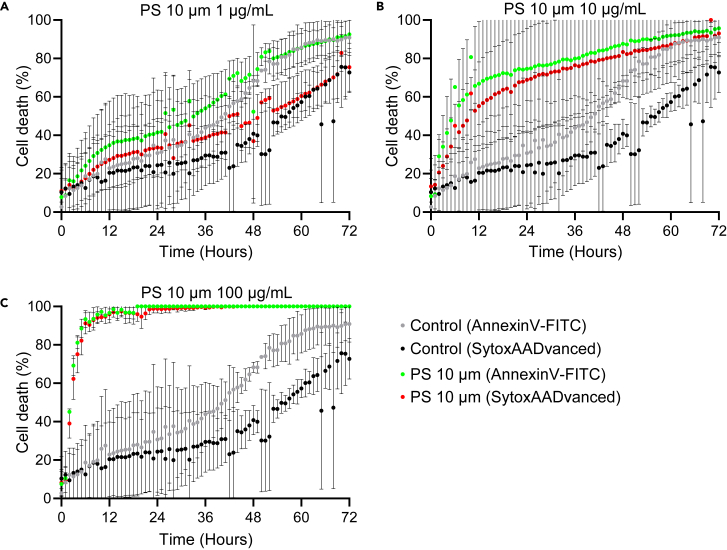
Neutrophil survival curves for PS 10 μm (A–C) The survival curves for the neutrophil control measured for AnnexinV-FITC (in gray) and SytoxAADvanced (in black). The survival curves for neutrophils exposed to PS 10 μm particles are visualized in green (AnnexinV-FITC) and in red (SytoxAADvanced). The neutrophils were exposed to PS 10 μm in the following concentrations: 1 μg/mL (A), 10 μg/mL (B) and 100 μg/mL (C). The values after the maximum value (100%) have been adjusted to one hundred. The normalized neutrophil survival curves are shown with standard deviation as error bars and represent the mean of three biological replicates n = 3.

Exposure to PS or silica particles or addition of 0.5% Triton X-100 after 72 h always lead to maximum cell death. Neutrophils and monocytes that are not exposed to particles function as control conditions. Based on previous work in our group and this protocol, higher particle concentrations induces more cell death compared to conditions with lower particle concentrations.^1^ The Incucyte S3 survival images followed over time and the normalized survival curves of the AnnexinV-FITC and SytoxAADvanced make this visible in neutrophils **(**Figure 8, Methods Video S1: Neutrophil survival after exposure 10 μm PS) and monocytes (Figure S2: Overview of the Monocytes AUC and slope of first three hours, Methods Video S2: Monocyte survival after exposure 10 μm PS).

Methods Video S1. Neutrophil survival after exposure 10 μm PS, related to Expected Outcomes

Methods Video S2. Monocyte survival after exposure 10 μm PS, related to Expected Outcomes

In this protocol, we describe how to retrieve information on particle-induced cell death based on the Incucyte S3 survival images and subsequent analysis by measuring the area under the curve (AUC) and the slope of the first three hours. For PS 10 μm, exposure to 10 and 100 μg/mL and for silica 1 and 10 μm exposure to 100 μg/mL, negatively affects neutrophil cell survival (Figure 10). Similarly in monocytes, exposure to PS 10 μm 100 μg/mL or silica increases cell death compared to the control or 1 and 10 μg/mL. However, particle toxicity in monocytes is less distinct compared to neutrophils and not statistically significant (Figure S2: Overview of the Monocytes AUC and slope of first three hours). It should be noted that neutrophils are very sensitive cells and have a shorter life span in vitro compared to monocytes. The data described in this protocol show that different innate immune cells are differently affected by particle-induced toxicity. Furthermore, PS 0.20 μm and 1 μm induce negligible effects on cell survival compared to the PS 10 μm, even in a concentration of 100 μg/mL. The MNP toxicity screening described here, reveals that particle-induced cell death is not only dose dependent but also size dependent. This in contrast to small molecule toxicity testing where most of the results are only dependent on dose. Here, a particle of different size but of the same polymer, can induce different effects on toxicity although they are applied in similar mass-based doses. A PS particle of 10 μm in size is the equivalent of 1000 particles of 1 μm in size. This reveals that in particle toxicity screening, there is also an extra component of the size of the particle that plays an important role.

## Quantification and statistical analysis

### Step-by-step exporting and normalization of the raw cell survival Incucyte data text file to a normalized.xlsx file

**Timing: 3 h**

The exported Incucyte data needs to be normalized to the maximum value of each condition to allow comparisons between conditions **(Graphical abstract, step 4)**. In this section, we describe the use of an R script to help you to change the ‘raw’ data from a text file into normalized data in an excel file.

***Note:*** The normalization method described in the protocol does not adjust for ‘lowest’ value to 0%. Cells already interact with the particles prior to the first scan. Therefore, we consider cell death data at the start already as valuable percentage.

***Optional:*** The normalization described in this protocol can also be performed manually. To do this manually, skip step 1 and 2 and use the following formula for each column:

$$\frac{Value}{{Value}_{\max}}\times 100={Value}_{Normalized}\%$$

1. When you have exported the text file from the Incucyte, run the normalization script in RStudio script **(**Box 1) to change the file type from text to Excel and to ‘clean’ the table from headers and time and data columns. The R script in box 1 normalizes each column to 100% as the highest value.
  a. The output data of the R script can be directly copy pasted to GraphPad Prism for further (statistical) analysis.
    **CRITICAL:** RStudio may give problems with reading the data in text or Excel. This depends on the (language) settings or version of your computer. In this protocol, we use version: RStudio version [1] ‘2024.12.0.467’.
    **CRITICAL:** Always make sure that your values in text are considered as numerical values, otherwise RStudio cannot normalize the data. Solve this by changing the ‘.’ of a decimal value to ‘,’ or vice versa.
    **CRITICAL:** To normalize the data from a text file and save the data in an excel file. You need to install and load the “xlsx” RStudio package in RStudio, this is included in the script in box 1.
    ***Note:*** In the line: as.data.frame(lapply(Name_XXX[1:16], normalize))∗100. The numbers from 1 to 16 are used because this protocol describes 16 conditions and thus 16 columns in the dataset. Change this manually to the number of experimental conditions in your set-up.
    ***Optional:*** You can manually convert the text file to an excel file by pasting the text file data into an excel file or export the data from the ‘clipboard’ in the Incucyte S3 Software directly into excel. Adjust the RStudio script to open an excel file from ‘read.delim’ into ‘read.xlsx’. In that case, you also need to manually ‘clean’ the data from headers and time stamps in Excel.
    **CRITICAL:** If you paste the data directly into an Excel template as described in the optional step described above, you have to manually change the decimal from ‘.’ to ‘,’. We recommend doing this in the text file.
    ***Optional:*** If you want to have the lowest value considered as 0, then you can also normalize the data of the excel file in GraphPad, see steps 1b, c below.
    ***Optional:*** In this protocol, we attached the raw Incucyte S3 text files as supplementary text files for the total area of AnnexinV-FITC and SytoAADvanced in neutrophils or monocytes, see Data S1**Survival data for AnnexinV and Sytox**. The data presented in this protocol is based on the normalization of these raw data files.
  b. In GraphPad, press ‘Analysis’ and then click on ‘normalize’ under ‘Transform, normalize’ tab.
  c. You can then decide to consider 0% as the lowest value.
    ***Note:*** You can also normalize manually in case you want to consider the lowest value as 0%. Then, use this formula:
    $$\frac{\left(Value-{Value}_{\min}\right)}{{Value}_{\max}}*100={Value}_{normalized}\;\%$$
2. Run the script for normalization as described in **box 1** for each individual experiment and proceed to step 3.

***Note:*** In this protocol**,** the mean value of the total green or red channel area from one biological replicate is based on six images per condition (three images per well in duplicate). The high-throughput screening assay data described in this protocol is obtained from three biologically different donors in three independent experiments.

***Note:*** In the previous section, you obtained two text files. This means that you run the script for each biological replicate twice, once for the green channel and once for the red channel.

3. Open GraphPad to place the normalized data from the excel file into GraphPad.
  a. Create a new GraphPad file by selecting: ‘XY’.
  b. In ‘Data table’, add: ‘enter or import data in new table’.
  c. Go to ‘Options’ and select in X: ‘Numbers’.
  d. Select in Y: ‘Enter number of replicate values in side-by-side sub columns’.
  e. Enter the box: ‘Mean, SD, N’.
  f. In the created GraphPad file, add in the X column the amount of timepoints you have and give the columns their condition name.

***Note:*** Here, we used GraphPad Prism version 10.04.0 (621).

4. Copy each column from the normalized data from all your biological replicates in the separate excel files into the created GraphPad template described in step 3a-f.

***Note:*** Neutrophils exposed to PS 10 μm particles have survival graphs for both cell death stains depicted in Figure 8**:** Neutrophil survival curves for PS 10 μm. Monocytes exposed to PS 10μm particles have survival graphs for both cell death stains as depicted in Figure S1: Monocyte Survival Curves for PS 10μm.

***Note:*** In this protocol, we compare control without particle exposure to each condition based on three biological replicates.

***Note:*** In GraphPad, you can manually choose the data you want to visualize in one graph for the neutrophil survival curve and give it a color of your own preference.

**CRITICAL:** If 100% cell death is reached within 72 h and the signal fades away after this time point, it is essential to correct the data after the maximum value to 100. Otherwise, analyzation of the AUC will be an underestimation of the cell death.

**CRITICAL:** Some of the data points of the survival curves can be much lower or higher compared to other data points. In that case, go back to the Incucyte Software and check if the images at the specific time point were not in focus or revealed autofluorescence.

***Optional:*** You can also check the z-position on unfocused images if these have been visualized on an aberrant z-position. Under ‘Tools tab’ you select ‘Vessel Information, ‘Image Metrics’ and then select ‘Focus Position’. Check per time point if those not in focus had a different z position. In GraphPad, decide to exclude these value(s) by clicking with your right mouse and click on ‘exclude values’. The value will then become blue with ∗ at the end. This data point will not be shown in the survival curve or taken along for further analysis.

Box 1 **Normalization script used both for neutrophil and monocyte cell death quantification**

# Make sure xlsx package is installed and functions

install.packages("xlsx", dependencies = TRUE)

library(xlsx) # Make sure it's loaded

# Set working directory

setwd("X:/.../.../.../.../.../...")

# Read .txt file and skip first 6 rows, read decimals in "." in table

XXX <- read.delim("Name_XXX.txt", skip = 6, sep = "\t", dec = ".")

# Skip first three columns of the table

Name_XXX_II <- Name_XXX[,-1:-2]

# Normalize numeric columns to maximum value

normalize <- function(x) {(x) / (max(x, na.rm = TRUE))}

Name_XXX_Normalized <- as.data.frame(lapply(Name_XXX_II[1:16], normalize))∗100

#save normalized data as .xlsx file

write.xlsx(Name_XXX_Normalized, "X:/.../.../.../.../.../.../Name_XXX_Normalized.xlsx")

### Calculation of the AUC and the slope of the first 3 h

**Timing: 2 h**

When you have successfully normalized your data and copied the data into GraphPad, it is time to calculate the area under the curve (AUC) and the slope of the first three hours to analyze cell death for the high-throughput screening. In this section of the protocol, the steps explain calculating the AUC, slope as well as subsequent statistical analysis.

The area under the curve.

5. When you have the normalized survival curves for each biological replicate, you can calculate the AUC of each replicate in GraphPad.
  a. Duplicate the normalized survival curves to calculate the AUC and slope of the first 3 h for each biological replicate, so for n=3, you have to duplicate the same file 3 times.
    ***Note:*** If you calculate the AUC and the slope of the first 3 h of the first file, calculate these values from the mean of the three replicates. If you want to calculate these values per replicate, you have to manually exclude the other replicates.
    ***Note:*** If you calculate the slope of the first 3 h (time points 0–3 h), exclude the values after time point 3 h of the desired replicate column. Otherwise, these values will also be included in the calculation.
  b. Calculate the AUC by clicking on the ‘analysis’ tab in GraphPad and search for ‘area under curve’ under the subtab ‘xy’.
  c. Select the data of which you want to calculate the AUC and press ‘OK’.
    **CRITICAL:** Make sure to use the same endpoint for all conditions and experiments. Otherwise, the AUC value is affected by shorter or longer timespans.
    ***Note:*** In the protocol described here, the endpoint is 72 h.
    ***Note:*** Calculate the AUC for each biological replicate individually by excluding the columns from the other replicates (otherwise you will calculate the mean AUC of all biological replicates).
6. Calculate the slope of the first 3 h by clicking on the ‘analysis’ tab in GraphPad.
  a. Go to ‘Fit a curve with nonlinear regression’.
  b. Proceed under ‘Standard Curves to interpolate’ and choose ‘line’.

***Optional:*** You can also calculate the slope of the first three hours in Excel, see the steps below.

The slope of the first three hours (measured from time point 0 h–3 h).

**Figure 9:**
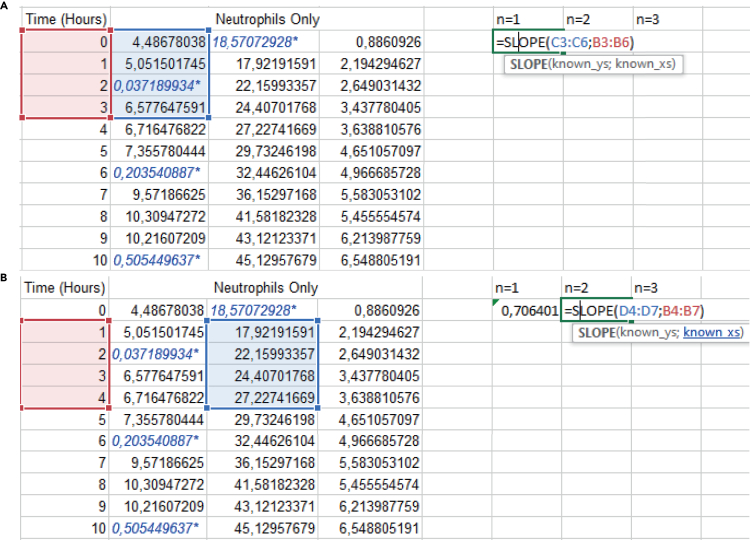
Calculation of neutrophil slope of the first three hours (time point 0–3 h) (A and B) Calculation of slope of three biological replicates with Excel formulas for n = 1 (A) and n = 2 (B). Note that for the first biological replicate the value at time point 2 h was not in focus and therefore excluded from the analysis (Blue and with asterisk on the end). For the second biological replicate, time point 0 h showed autofluorescence, therefore the slope was calculated from time point 1–4 h. The values in each column are based on the normalized mean of two technical replicates with three images each from the Incucyte Analysis Software. Each column represents the mean of one biological replicate (n = 3).

7. Manually copy the normalized values from GraphPad in a new Excel file and determine the slope of the first three hours of each biological replicate individually (here n = 3).
  a. You calculate the slope of the first four time points (time points 0–3 h) of the normalized curve in Excel file by the following function =SLOPE(known_ys; known_xs) (Figure 9A).
  b. Determine the slope of each individual biological replicate (here n = 3).

**CRITICAL:** Sometimes, the first time point (0 h) is not in focus and needs to be excluded. In that case, calculate the slope for time point 1–4 h instead of 0–3 h (Figure 9B). If other time points are not in focus, for example the time point 1 or 2 h, you can still calculate the slope based on the time point 0 h and the time point 3 h. You can also consider calculating the slope of the first five time points (0–4 h) instead of the first four time points.

***Note:*** If more than one time point needs to be excluded, decide if you can still use the survival curve to calculate the slope of the first four or five hours or to exclude the whole replicate.

**CRITICAL:** We chose the slope of the first four time points because we observed that the particle effects are the strongest during the first time points during particle exposure. You have to decide for yourself on which time points you want to base the initial slope. When data points in the initial hours are blurry you can decide whether you calculate the slope of time point 1–4 h. If you wait too long or extent the number of first time points, you may miss the increase in cell death signal because it has already ‘happened’. In that case, the slope may flatten because of the later time points.

***Note:*** You can also perform a curve fit in GraphPad of the first time points which calculates the slope, depending on the time points you want to measure this.

***Note:*** The curve fit for the full 72-h survival curve flattens the curve which understates the effects of particles on cell viability. Therefore, we look at the initial time points because the later time points do not add more to the cell death assessment if quantified via the slope of the curve.

8. Copy and paste values of the AUC and slope in a new GraphPad file to perform additional statistical analysis.

**Figure 10:**
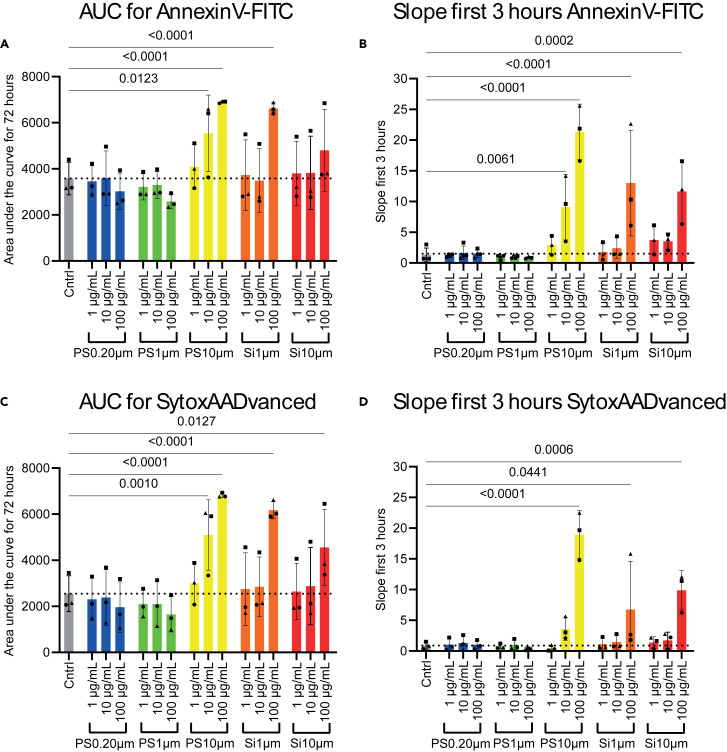
Overview of the calculated neutrophil area under the curve and slope (A–D) Neutrophil area under the curve (AUC) for 72 h (A) and slope of the first three hours (B) for AnnexinV-FITC and the AUC (C) and slope (D) for SytoxAADvanced. Neutrophil control visualized in gray, polystyrene (PS) 0.20 micrometer (μm) in blue, PS 1 μm in green, PS 10 μm in yellow, silica 1 μm in orange and silica 10 μm in red. Dotted line represents the mean of the control. Data is represented as mean including standard deviation. Each symbol represents one biological replicate (n = 3).

***Note:*** An overview of neutrophil AUC and initial slope of the survival curves after particle exposure are shown in Figure 10. For monocytes, the AUC and the slope of the first 3 h of the survival curves after particle exposure can be found in Figure S2: Overview of the Monocytes AUC and slope of first three hours.

9. For both the AUC and slope of the first three hours, you want to calculate in GraphPad if there is a statistically significant difference between the three biological replicates as performed in this protocol.
  a. To do this in GraphPad, select ‘Analyze’, use one-way (and nonparametric or mixed) ANOVA.
  b. Select only your control and the values you want to compare.
  c. Since data described in this protocol is matched, choose: ‘Each row represents matched or repeated measures data’.

***Note:*** You can only perform paired statistical analysis if every condition has the same replicates and numbers. Otherwise, you have to test without ‘matching or pairing’. You can then continue to step 10a-c.

10. Prepare the settings for statistical analysis of the data.
  a. Assume Gaussian distribution, select for ‘Yes, use ANOVA’.
  b. ‘Assume sphericity’ and select for ‘Yes, no correction’.
  c. Go to the tab ‘multiple comparisons’ and select ‘Compare the mean of each column with the mean of a control column’.

***Note:*** In this protocol, every condition was measured from the same biological sample. You have to adjust the statistical settings for your own experimental set up.

***Note:*** In this protocol, we consider the data statistically significant when p < 0.05.

***Note:*** The neutrophils and monocytes are primary cells from different donors. Therefore, there is high donor variability in toxicity effects.

**CRITICAL:** You always need a minimum of three independent experiments to perform statistical analysis.

## Limitations

The Incucyte S3 is only suitable if you are interested in experiments where you visualize the phase-contrast and green (excitation between 440–480 nm and emission between 504–544 nm, e.g., FITC, GFP, CaleinAM) or red (excitation between 565–605 nm and emission between 625–705 nm, e.g., SytoxAADvanced or propidium iodide) fluorescence. Fluorophores that have another wavelength excitation/emission are not suitable for the Incucyte S3. If you use all three channels (Phase, Green and Red) in a full plate and image many images per well, there is a chance that you exceed more than 30 min per hour of scanning time. The Incucyte S3 can only image for a maximum time of 30 min per hour. If you exceed the scanning time, this will cause an error, so you need to balance the optimal number of images and channels you want to capture. Besides, check with other plates and experiments in the Incucyte S3.

The protocol as described here, is only applicable when you seed a monolayer of cells. The Incucyte S3 cannot image any cells in a higher plane other than the bottom. Furthermore, cells or particles in a higher plane might hinder the analysis by creating background interference or images not in focus.

The plating of the monocytes as described here, will be an estimation because we do not calculate the monocytes directly but estimate their count by dividing the white blood cells after isolation of the PBMC layer by 5. However, different donors do have different monocyte counts.

You can quantify cell death from the survival graphs in diverse ways. Here we use quantification of the area under the curve (AUC) and the slope for the first three hours. Another method to investigate cell death followed over time, is to calculate the timepoint where 50% of cell death is reached. However, monocytes are not as affected by particles as compared to neutrophils. In our hands, monocytes sometimes do not reach 50% cell death within 72 h so you cannot use that method to quantify cell death for the described particles within this time frame.

From the moment that you mix the particles and neutrophils in the Eppendorf; cells will be able to engulf plastic particles and potentially start dying. Although we work as streamlined as possible, it will not be possible to detect the first dying cells that are affected by the particles.

Another limitation in this study is that we did not include a non-particle positive cell death control. The time upon how quick 100% cell death is reached, tells you something about the toxicity of a particle compared to a cell death control. One could include a condition where 20 μl of 0.5% Triton X-100 is added and mixed in the well at the start of the experiment to induce immediate cell death. Addition of Triton X-100 leads to quick rupture of the cell membranes and immediate cell death. You can add this as a positive control to compare how quickly cell death occurs. Other cell death inducers can also tell you more about the specific forms of cell death.

In this protocol, we did not specify what form of cell death the polystyrene and silica particles induce. In earlier studies, AnnexinV-FITC is used as an early onset cell death marker because an impaired membrane integrity is one of the first hallmarks of a dying cell.^12^ SytoxAADvanced is a cell death marker that binds to nucleic acids of DNA in the cell nucleus.^13^ This is only possible when the integrity of the cell membrane is gone and therefore should happen after AnnexinV binding. This is comparable to AnnexinV/Propidium Iodide staining.^14^ In the high-throughput screening assay described here, we observe that dual staining rapidly occurs. If apoptotic death occurs, the extracellular phosphatidyl serine stained by AnnexinV-FITC is expected to precede cell permeability and DNA staining by SytoxAADvanced. However, the clumping of cells in our assay specifically upon microplastic exposure makes this difficult to distinguish because signals of different cells overlap (Methods Video S1: Neutrophil survival after exposure 10 μm PS). This clumping also made it impossible to mask for NETotic or pyroptotic cells which form a large area DNA outside the cell which interferes with the cell clumping. If you want to properly distinguish between apoptosis and necrosis or other specific forms of cell death (for example NETosis or pyroptosis), we recommend seeding the cells at a lower density or use more detailed microscopy techniques with higher magnification and live measurements at higher intervals. However, the advantage of the Incucyte S3 microscope and corresponding software is that it can quickly measure many different conditions at a desired intervals for longer periods of time.

## Troubleshooting

In this section, we describe the problems that can arise by following this high-throughput screening protocol for neutrophils and monocytes. Success of this high-throughput cell death screening depends on the quality of the imaging. You can only analyze bright, clear, and focused time lapse images. Therefore, the solutions described in this section are based on optimizing the conditions and visualization of the images when this is not the case.

### Problem 1

The cells are dying directly after plating because of the cell/particle interactions.

### Potential solution

• Work as streamlined as possible to reduce time loss. If this is not working, you can try to add the neutrophils first in 100 μL in the plate instead of the Eppendorf and then later add the rest of the particles to minimize the initial interaction. In that case, you still have to apply the Incucyte S3 settings before you can initiate a timelapse measurement which takes time.
• When you have a lot of conditions in the plate, some of the neutrophils in the conditions can already interact with the particles form the conditions you already pipetted. If this is a problem, decide to remove some conditions to improve streamlining the experiment.

### Problem 2

**Figure 11:**
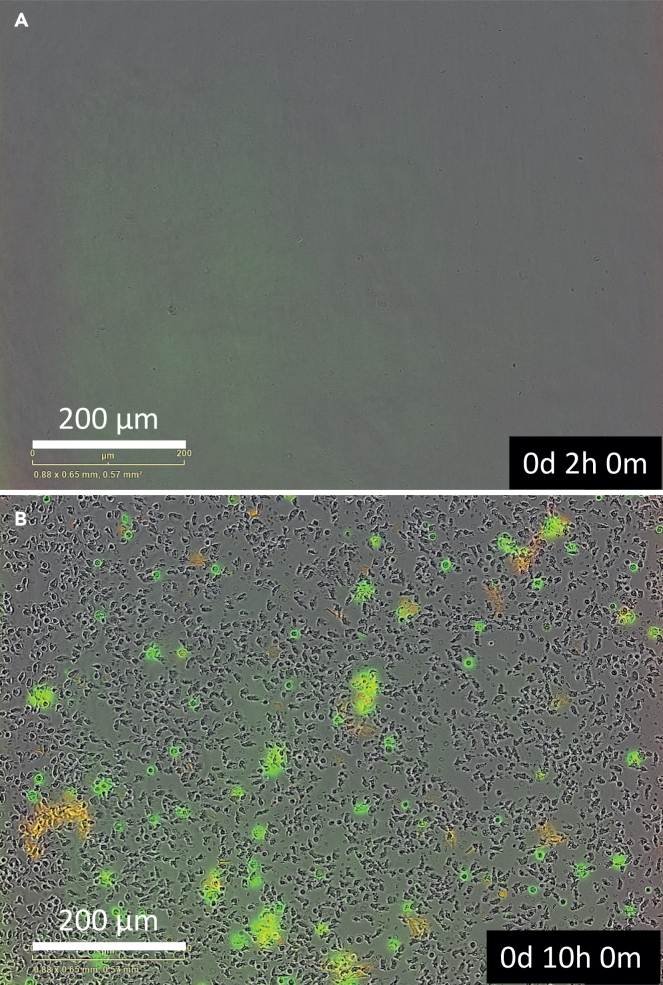
In and out focus image of the same well at different time points (A and B) The Incucyte S3 could not find the focus position and only green background signal is present at time point 2 h (A). The Incucyte could find focus position for the cells at time point 10 h (B). The image is made with a 20× magnification of the neutrophil only condition where cell death is visible as AnnexinV-FITC staining in green and as SytoxAADvanced in orange. Dual stained cells are visible in a yellow merge color. The scale bar represents 200 μm.

The Incucyte cannot find the correct focus position. This is the case when the image is blurry or has much auto fluorescent signal (Figure 11).

### Potential solution

• One explanation of a blurry image is that you image the plate too quickly after adding the conditions to the plate, see step 13 under setting up the Incucyte S3 instrument for visualization of cell death over time. The neutrophils and particles need a minimum of 15 min to sink to the bottom of the plate. If the cells and particles are still in suspension and sink, the Incucyte S3 may have problems with focusing.
• Out of focus images can be caused by expansion of the plate because of the transfer at 21°C (room temperature) to the 37°C in the Incucyte incubator. Overcome this problem by preincubating the plate 15 min in the Incucyte S3 before the start of the measurement to make sure that the plate is adjusted to the different temperature. Usually, it is sufficient to preincubate the plate in the Incucyte before the measurement when you are adjusting the settings. We always recommend checking if the first images are in focus.
• To solve focusing problems by the Incucyte, add the positive control in the first two wells of the 96-wells plate, add 20 μL of 0,5% Triton X-100 directly after pipetting all the conditions in the wells as prepared in step 20. This will quickly cause permeabilization of the cell membrane to induce cell death and subsequent staining with AnnexinV-FITC. This improves contrast so that the Incucyte S3 can improve its focus, as the largest focus sweep of the Incucyte is performed on the first scanned well, you cannot use these wells in the analysis to quantify cell death over time.

### Problem 3

Condensation is forming on the lid of the plate in the IncuCyte S3 and is disturbing the focus.

### Potential solution

When pipetting all conditions into the plate, keep it at 21°C and not cool it down at 4°C. Also for this problem, the 15 min waiting time in the incubator 37°C of the IncuCyte S3 will help.

### Problem 4

After pipetting all the mixtures in the plate, it might be the case that bubbles are present on top of the medium. The Incucyte S3 can have trouble with focusing if bubbles are present.

### Potential solution

If bubbles are persistent you can try to pinch them with a sterile pipet tip or pop them with a sterile needle. If this does not help, you can also try to remove bubbles with a sterile and empty wash bottle by blowing out air over the well with bubbles. The air can blow away the bubbles.

### Problem 5

The RStudio script worked before but stopped working after a software update.

### Potential solution

Install and load the packages again after an R studio update. Also make sure that the adequate Rtools and Java software version is installed and up to date, see step 1 under the Step-by-Step exporting and normalization of the raw cell survival Incucyte data text file to a normalized .xlsx file.

Although not ideal, you can always manually normalize the data in Excel.

### Problem 6

High backgrounds make it difficult to correctly mask the cell death in the Incucyte S3 analysis software.

### Potential solution

If this problem cannot be solved by masking during the analysis, it might be helpful to use RPMI 1640 medium without phenol red for your cells to prevent background. The HEPES3+ buffer is colorless. The problem with incorrect masking of the background may be overcome by using a different masking setting. The Top Hat segmentation may cause problems and includes a lot of background during masking. Therefore, we recommend always using Surface Fit which is also the default setting in the Incucyte S3 analysis software.

## Resource availability

### Lead contact

Further information and requests for resources and reagents should be directed to and will be fulfilled by the lead contact, Nienke Vrisekoop (n.vrisekoop@umcutrecht.nl).

### Technical contact

All technical questions related to the Ficoll isolation and experimental settings should be directed to and will be fulfilled by the technical contact, Nienke Vrisekoop (n.vrisekoop@umcutrecht.nl).

### Materials availability

This study did not generate new unique reagents.

### Data and code availability

• Further information and request for the data and the code described in this protocol will be fulfilled by the lead contact, Nienke Vrisekoop (n.vrisekoop@umcutrecht.nl).
• The datasets supporting the current protocol have not been deposited in a public repository, but they are available from the corresponding author on request.

## Acknowledgments

This study was supported by the POLYRISK project, funded by the 10.13039/100010661EC Horizon 2020 program under GA no. 964766 and by the MOMENTUM project (project number 458001101), and has been made possible by 10.13039/501100001826ZonMw Programme Microplastics and Health and Health-Holland, Top Sector Life Sciences & Health.

## Author contributions

T.L.P.S., S.S., J.A.Z.K., G.G., and N.V. conceived of and designed the experiments. T.L.P.S., S.S., and L.L.F.H. performed the experiments. T.L.P.S. set up the analysis parameters, reviewed by J.A.Z.K. and N.V. J.A.Z.K. and T.L.P.S. wrote the original manuscript. N.V. reviewed and edited the manuscript. N.V. acquired funding and supervised the project. All authors contributed to the manuscript**.**

## Declaration of interests

The authors declare no competing interests.

## Supplemental information

Document S1. Figures S1 and S2

Data S1. Survival data for AnnexinV and Sytox
