# Supplementary material for: Protocol for dynamic high-throughput cell death screening of primary phagocytes following microplastic and nanoplastic exposure
Source: STAR Protoc. 2025 Nov 14;6(4):104204. doi: 10.1016/j.xpro.2025.104204 (PMC12664042; doi:10.1016/j.xpro.2025.104204)
Supplement: Document S1. Figures S1 and S2 [file mmc1.pdf]

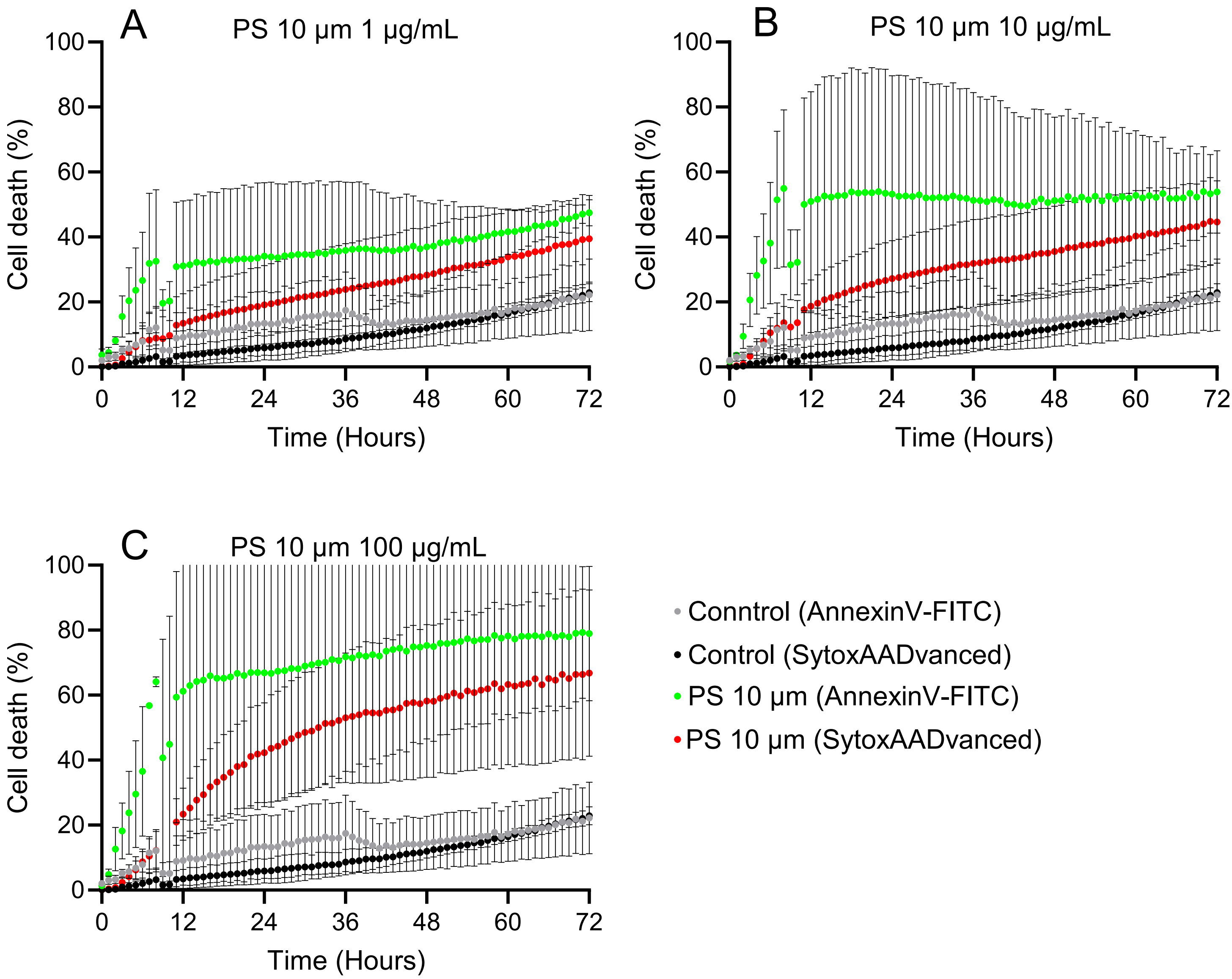

Supplementary Figure 1. Monocyte survival curves for PS 10 µm, related to step 4 in quantification and statistical analysis. The survival curves for the monocyte control measured for AnnexinV-FITC (in gray) and SytoxAADvanced (in black). The survival curves for monocytes exposed to PS 10 µm particles are visualized in green (AnnexinV-FITC) and in red (SytoxAADvanced). The monocytes were exposed to PS 10 µm in the following concentrations: 1 µg/mL (A), 10 µg/mL (B) and 100 µg/mL (C). The values after the maximum value (100%) have been adjusted to one hundred. The normalized monocyte survival curves are shown with standard deviation as error bars and represent three biological replicates n = 3.

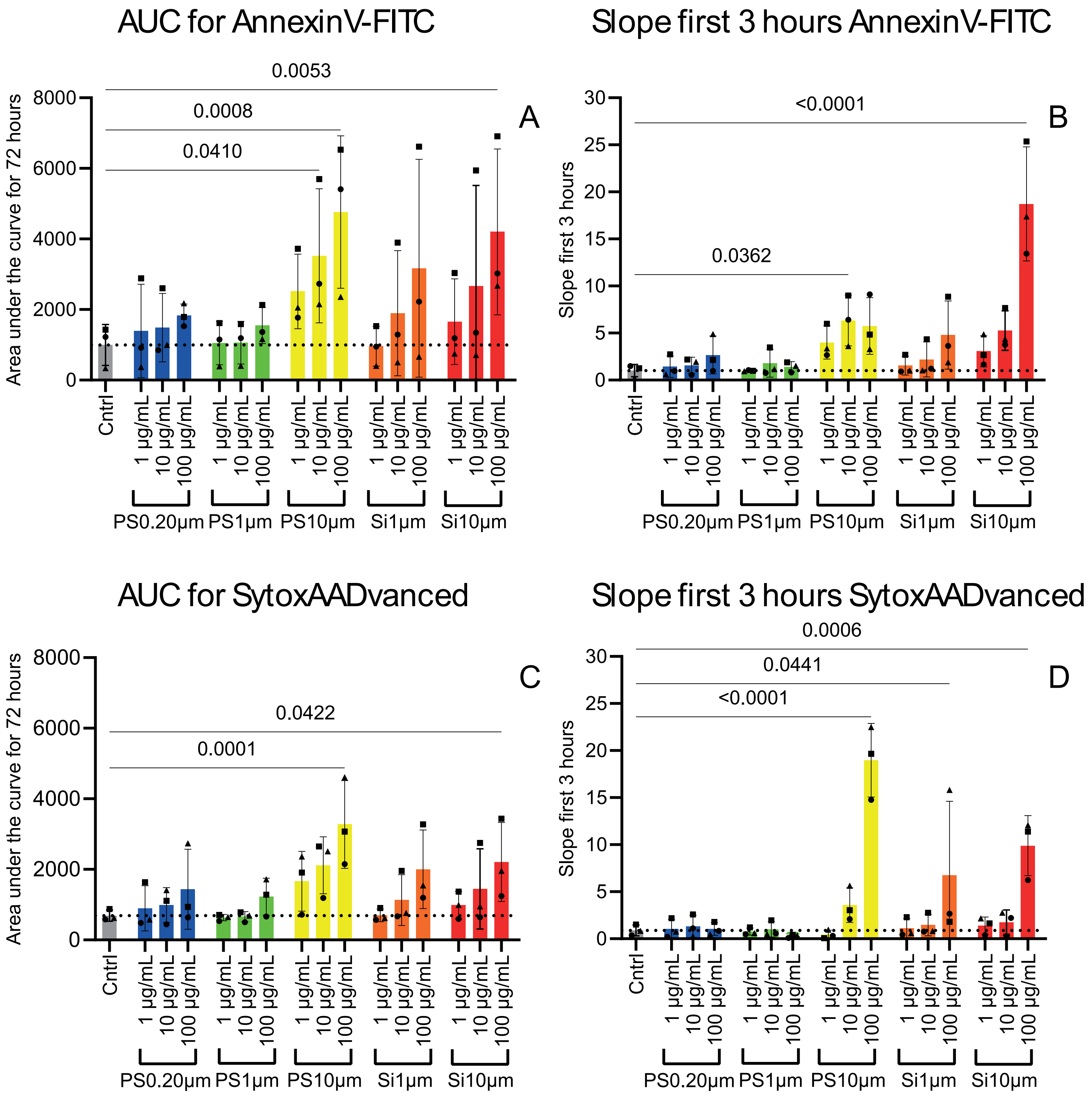

Supplementary Figure 2. Overview of the monocyte area under the curve and slope of the first three hours, related to step 8 in calculation of the AUC and slope of the first 3 hours. Area under the curve (AUC) for 72 hours (A) and slope of the first three hours (B) for AnnexinV-FITC and the AUC (C) and slope (D) for SytoxAADvanced. Monocyte control visualized in gray, polystyrene (PS) 0.20 µm in blue, PS 1 µm in green, PS 10 µm in yellow, silica 1 µm in orange and silica 10 µm in red. Dotted line represents the mean of the control. Each symbol represents one biological replicate (n = 3).
